# Revisiting oxygen toxicity: evolution and adaptation to superoxide in a SOD-deficient bacterial pathogen

**DOI:** 10.1128/mbio.00645-25

**Published:** 2025-07-23

**Authors:** Samuel G. Huete, Alejandro Leyva, Etienne Kornobis, Thomas Cokelaer, Pierre Lechat, Marc Monot, Rosario Duran, Mathieu Picardeau, Nadia Benaroudj

**Affiliations:** 1 Biology of Spirochetes, Institut Pasteur, CNRS UMR 6047, Université Paris Cité 555089, Paris, France; 2 Analytical Biochemistry and Proteomics Unit, Instituto de Investigaciones Biológicas Clemente Estable, Institut Pasteur de Montevideo 123939, Montevideo, Uruguay; 3 Plate-forme Technologique Biomics, Institut Pasteur, Université Paris Cité 555089, Paris, France; 4 Bioinformatics of Biostatistics Hub, Institut Pasteur, Université Paris Cité 555089, Paris, France; McGovern Medical School, Houston, Texas, USA

**Keywords:** *Leptospira*, spirochetes, superoxide dismutase, oxygen toxicity, sulfur assimilatory pathway, regulation of gene expression, evolution, RNA-seq

## Abstract

Dioxygen (O_2_) is vital for aerobic life, but its utilization leads to the inevitable production of superoxide, a toxic oxidant. The prevailing theory of oxygen toxicity postulates that superoxide-scavenging enzymes (SOSEs), such as superoxide dismutases (SODs), are crucial for most aerobes and play a key role in the virulence of pathogens. However, our knowledge of superoxide adaptation primarily stems from the study of SOSE-encoding bacteria. Here, we investigated the evolution of a naturally SOSE-deficient pathogen (*Leptospira* spp.) and its alternative mechanisms to combat superoxide stress. We demonstrated that SOD was ancestral in the genus *Leptospira* but lost by pathogenic species, and heterologous expression of a SOD in this pathogen did not improve superoxide tolerance. In *L. interrogans*, inheritable increased expression of a genetic locus, including a MFS transporter, mediated a long-lasting adaptation to superoxide, independently of any permanent genetic modification. Using a multi-omics approach, we identified a *leuA2*-encoded isopropylmalate synthase, the enzyme catalyzing the first step of leucine biosynthesis, as the most upregulated factor by superoxide. Interestingly, LeuA2 lacks the canonical domain for feedback inhibition by leucine and is the only upregulated factor of leucine biosynthesis, suggesting a moonlighting activity for LeuA2 in the adaptation to superoxide. Moreover, the cysteine biosynthesis pathway was significantly upregulated in response to superoxide, and we demonstrated the importance of sulfur metabolism in adaptation to superoxide. This study revisits our conventional understanding of the oxygen toxicity theory and proposes a new model of superoxide adaptation through redox-based metabolic rewiring in SOSE-deficient aerobic bacteria.

**IMPORTANCE:**

Superoxide is a toxic reactive oxygen species produced as an inevitable byproduct during oxygen respiration. It is therefore assumed that aerobic bacteria require superoxide scavenging enzymes (SOSEs), such as superoxide dismutases. Recent studies estimate that around 10% of all living organisms lack SOSEs. However, we ignore how these organisms survive superoxide stress when confronted with oxygen. Here, using *Leptospira interrogans*, a naturally SOSE-deficient aerobic pathogen, we address the evolutionary path and defense mechanisms leading to the adaptation to superoxide in the absence of any SOSE. We demonstrate that a SOD was ancestral in this genus but was lost with the emergence of pathogenic species. In addition, we show that pathogenic *Leptospira* induce metabolic pathways to fight superoxide, such as cysteine biosynthesis and isopropylmalate synthase. Thus, our study reveals that redox-based metabolic reprogramming may compensate for the loss of SOSEs in pathogenic bacteria.

## INTRODUCTION

Superoxide anion (O_2_^•−^) is an oxidant resulting from the one electron-reduction of dioxygen (O_2_) in living organisms. Aerobic pathogens not only encounter the superoxide generated during their own oxygen metabolism but also high concentrations of host-produced superoxide during infection. Superoxide is spontaneously converted into hydrogen peroxide (H_2_O_2_), but this reaction can be catalyzed by superoxide-scavenging enzymes (SOSEs) like superoxide dismutases (SODs) or reductases (SORs) 10,000 times faster (1). SOSEs are thus considered as the first line of defense against the toxic effects of superoxide, allowing its rapid elimination from the bacterium.

The oxygen toxicity theory, formulated more than 4 decades ago, posits that (i) superoxide is a toxic molecule produced within aerobic organisms and (ii) SODs (and SOSEs in general) are vital for aerobic organisms to detoxify O_2_^•−^ (2, 3). However, this theory was established by studying model bacteria and has not been extensively examined across various microorganisms, overlooking SOSE-deficient pathogenic aerobes. How these aerobic bacteria protect themselves against superoxide remains to be elucidated.

The genus *Leptospira* includes both free-living saprophytes as well as pathogenic species responsible for leptospirosis, a re-emerging yet often neglected zoonotic disease (4). Leptospires are aerobes exposed to endogenous ROS, but only pathogenic species can survive the oxidative stress imposed by the host’s innate immunity (5). Specifically, pathogenic *Leptospira* spp. possess a catalase that efficiently degrades H_2_O_2_ and is required for *Leptospira* virulence (6, 7). Previous genomic comparisons within the *Leptospira* genus suggested that certain pathogenic species may lack any SOD-encoding gene (8), but a large-scale comprehensive analysis was required to confirm this finding.

Here, we investigate the universality of the oxygen toxicity theory to all human bacterial pathogens. We present evidence challenging its assumptions, by showing that two aerobic pathogens (*Leptospira* spp. and *Mycoplasmataceae* spp.) lack conventional superoxide detoxification systems. We also deciphered the evolutionary events behind the loss of SOD in pathogenic *Leptospira* spp. Using a multi-omics approach, we demonstrated that superoxide adaptation remains possible in the absence of SOSEs and characterized the role of cysteine and leucine biosynthesis as the most induced pathways in response to superoxide. Altogether, our findings expand our understanding of how aerobic pathogens respond to oxidative stress, encouraging us to revisit the assumptions of the oxygen toxicity theory.

## RESULTS

### Distribution of SOSEs among bacteria

**Fig 1:**
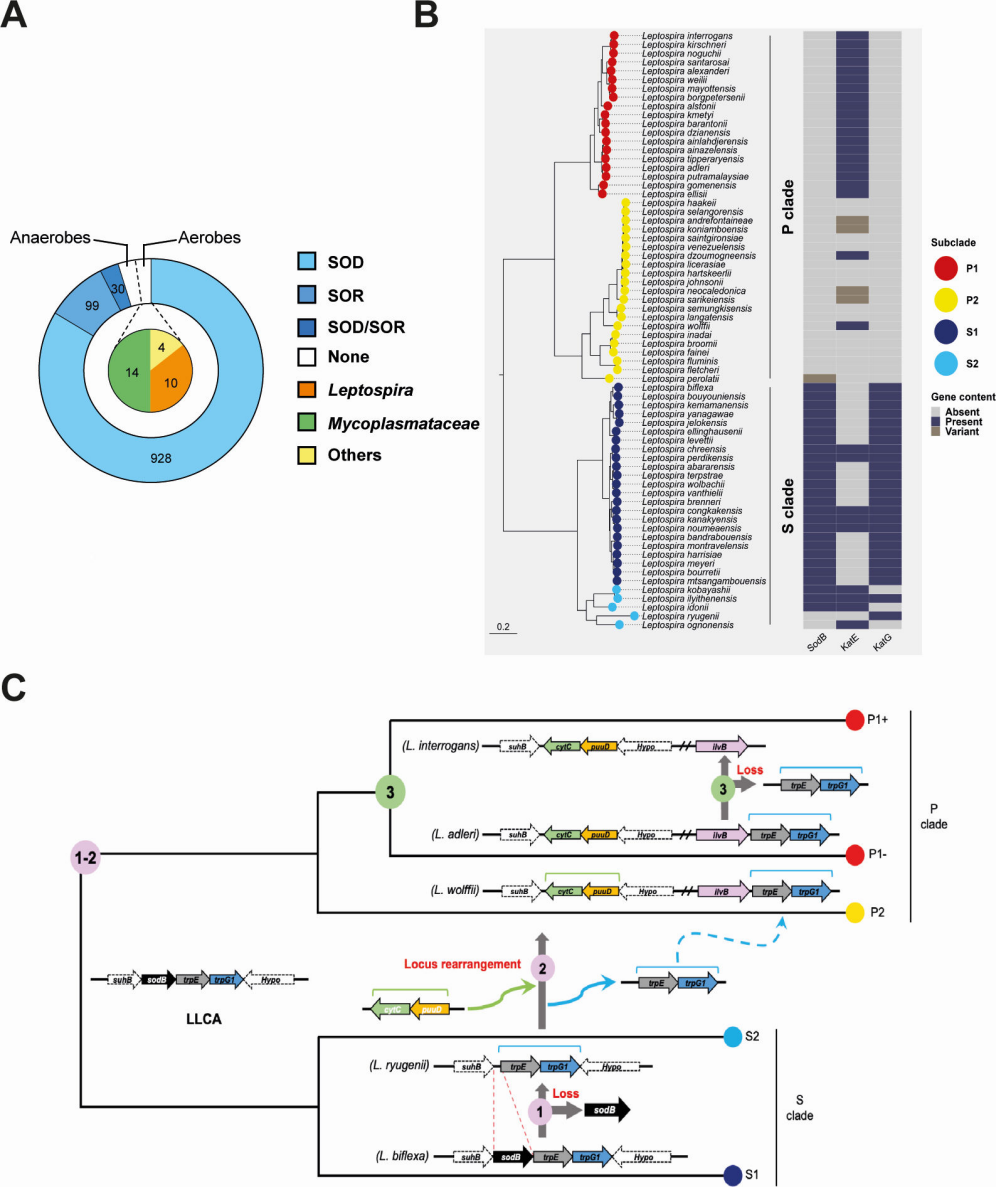
Pathogenic *Leptospira* spp. have lost their SOD. (**A**) Distribution of SOSEs (SOR or SOD) in different bacterial pathogenic species. SOD and SOR orthologs were searched in a pathogen genome database using a e-value cutoff of 0.01. Aerobicity was indicated for the species that do not contain any SOSE. Numbers correspond to the respective number of genomes. (**B**) Phylogenetic distribution of SOD and catalases (KatE or KatG) in 68 *Leptospira* spp. with an e-value cutoff of 0.01. P1 and P2 species (P clade) are in red and yellow, respectively. S1 and S2 species (S clade) are in dark blue and cyan, respectively. Blue: present, light gray: absent, dark gray: variant. (**C**) Schematic evolutionary trajectories leading to the loss of SOD in pathogenic *Leptospira* spp. The *sodB* loci are represented in the Last Leptospiral Common Ancestor (LLCA), in S1 (dark blue), S2 (cyan), P2 (yellow), P1− (low virulent), and P1+ (high virulent) (red) species. The three key steps are indicated.

To gain a comprehensive understanding of the prevalence of SOSEs in pathogenic bacteria, we analyzed a database of 1,110 established bacterial pathogens infecting humans (9). We identified that over 95% (*N* = 1057) of bacterial pathogens encode a SOSE, whereas less than 5% (*N* = 53) lack any SOSE in their genome (Fig. 1A; Table S1). Surprisingly, we found a nearly equal distribution between aerobes and anaerobes among SOSE-deficient species. Most aerobic SOSE-deficient pathogens are in the *Mycoplasmataceae* family and the *Leptospira* genus (Fig. 1A). *Mycoplasmataceae* spp. have reduced genomes (0.58–1.73 Mb) and metabolism, including the absence of oxidative phosphorylation (10). However, *Leptospira* spp., with a complete respiration chain capable of generating superoxide (11), offer a suitable model for investigating adaptation to superoxide in SOSE-deficient aerobic pathogens.

### Pathogenic *Leptospira* lost the *sod* gene in the evolution toward pathogenicity

To decipher the evolutionary trajectories leading to the appearance of SOSE-deficient species in *Leptospira*, we investigated the evolution of the SOD and SOR-encoding genes in the *Leptospira* genus and the *Spirochaetes* phylum. This phylum comprises several pathogens in addition to *Leptospira*, such as the causative agents of Lyme disease, relapsing fever, and syphilis (*Borrelia burgdorferi*, *Borrelia hermsii*, and *Treponema pallidum*, respectively). We constructed a core-genome phylogeny of the 164 culturable species of the *Spirochaetes* phylum and mapped the presence of a SOD or SOR-encoding ORF (Fig. S1; Table S2). Our findings revealed that 72% (*N* = 118) of *Spirochaetes* encode a SOSE. The presence of a SOR does not consistently correlate with the absence of a SOD but it does with anaerobicity (66% of anaerobes have a SOR) (Fig. S1). Among the 28% (*N* = 46) of *Spirochaetes* lacking any SOSEs, 89% (*N* = 41) are *Leptospira* spp., the remaining five being strict anaerobes (Fig. S1). Hence, pathogenic *Leptospira* spp. are the only aerobic *Spirochaetes* lacking a SOSE-encoding gene.

Intriguingly, although most *Leptospira* spp. from the P clade, containing pathogenic species, lack any protein with homology to SOD, a *sod* gene is present in almost all species from the S clade, containing strictly environmental saprophytic species (Fig. 1B; Fig. S2). Evolutionary analysis revealed that the Last Leptospiral Common Ancestor (LLCA) possessed a *sodB* gene, which was maintained in S clades and subsequently lost when *Leptospira* transitioned from S to P clades (Fig. 1C; Fig. S1 and S2). Consistently, all *sodB* genes in *Leptospira* spp. exhibit a highly conserved genetic locus, whereas the *sodA* homolog in *L. perolatii* was very likely acquired independently through horizontal gene transfer (Fig. S2). Genus-wide comparative genomic analysis of the *sodB*-encoding locus revealed that *trpE* and *trpG1*, the two genes downstream *sodB*, were displaced downstream *ilvB* and substituted with *puuD* and *cytC*. This locus rearrangement probably occurred subsequently to the loss of *sodB* as it is possible to identify S2 species (such as *L. ryugenii*) where *trpE* and *trpG1* are present in their original loci despite the absence of a *sodB* (Fig. 1C; Fig. S2). The *sodB* loss and *trpE* and *trpG1* displacement likely occurred before the emergence of the P clade (steps 1-2 in Fig. 1C; Fig. S2). Finally, *trpE* and *trpG1* were lost by deletion upon the transition of P1- group (species with low virulence) to the P1+ group (highly virulent species) (step three in Fig. 1C; Fig. S2). Altogether, these findings not only establish that *Leptospira* is one of the few SOSE-deficient aerobic pathogens but also reveal that the loss of its SOD coincided with the transition from saprophytes to host-adapted species.

### Heterologous expression of *sodB* in *L. interrogans* impairs fitness in the presence of superoxide

**Fig 2:**
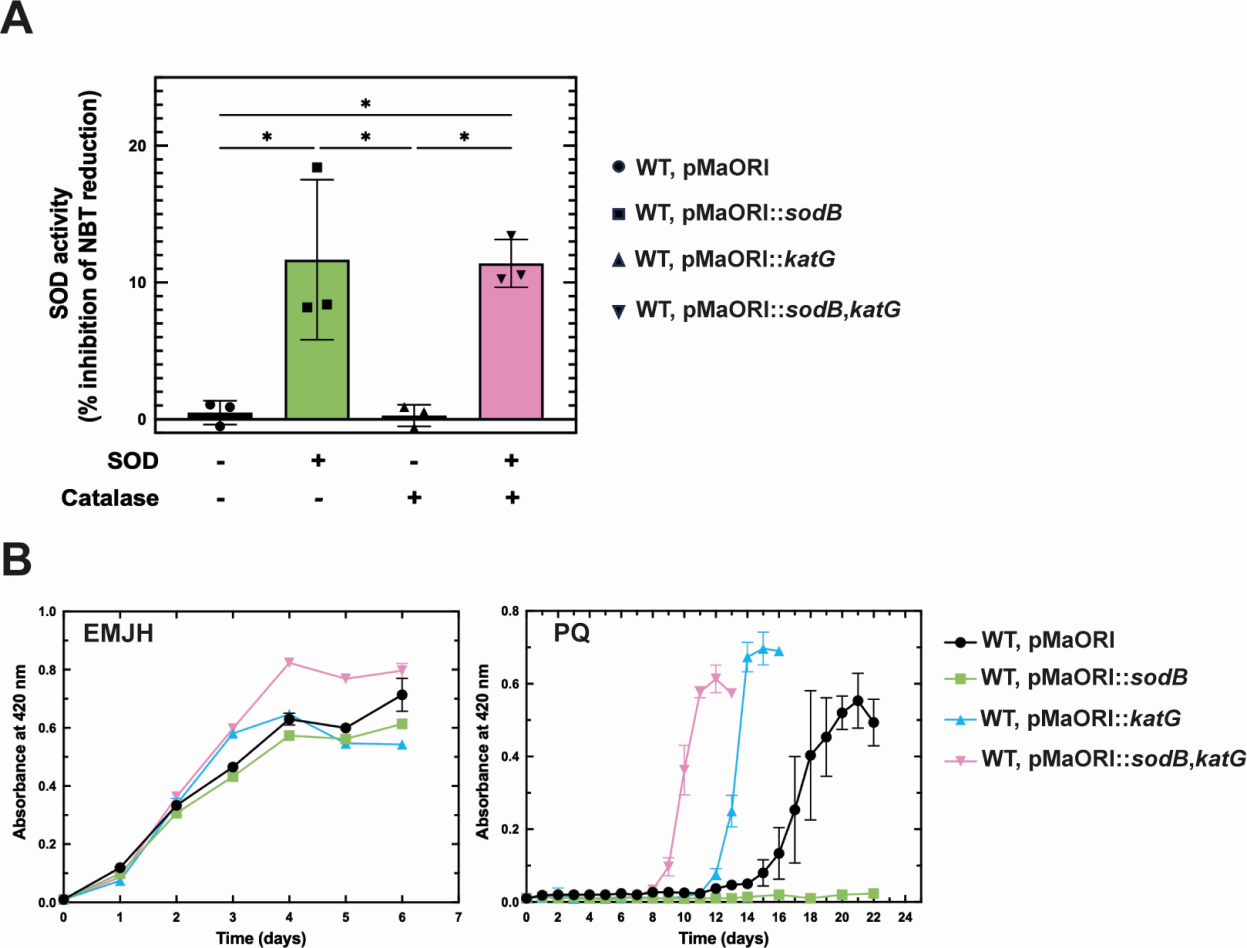
Expression of sodB in *L. interrogans*. (**A**) SOD activity in the WT (circle), *sodB*-expressing (square), *katG*-expressing (triangle), and *sodB*- and *katG*-expressing (inverted triangle) *L. interrogans* strains. The activity is expressed as the percentage of NBT reduction inhibition per μg of total extract. Data are the mean and SD of three independent experiments. Statistical analysis was done by a one-way ANOVA test with Bonferroni’s multiple comparisons test (*, *P*-value of 0.01). (**B**) Growth curve of WT (black circle), *sodB*-expressing (green square), *katG*-expressing (blue triangle), and *sodB*- and *katG*-expressing (pink inverted triangle) *L. interrogans* strains in the absence or presence of 2.5 µM paraquat. Growth was assessed by measure of absorbance at 420 nm. Data are the mean and SD of three independent experiments.

Given that pathogenic *Leptospira* have lost *sodB*, we tested whether the presence of a SOD activity would improve their tolerance to superoxide. The *sodB* gene of *L. biflexa* was expressed and a SOD activity could be detected in *L. interrogans* (Fig. S3; Fig. 2A). Surprisingly, the presence of an active SOD impaired *L. interrogans* growth in the presence of paraquat, a well-known superoxide generating compound (Fig. 2B). We ratiocinated that the introduction of an active SOD in *L. interrogans* would increase the cytoplasmic H_2_O_2_ content produced consequently of superoxide dismutation. *L. interrogans* only have one catalase (KatE) located in the periplasm (6, 12) (Fig. 1B). We therefore tested whether the presence of a cytoplasmic catalase (KatG), together with SOD, would improve the *L. interrogans* growth in the presence of paraquat. Expression of the *L. biflexa* catalase-encoding *katG* in the *sodB*-expressing *L. interrogans* strain rescued its ability to grow in the presence of paraquat (Fig. 2B). *KatG* expression also reduced the lag phase and increased the growth rate of the WT strain, which does not express *sodB*, in the presence of paraquat. This difference in tolerating paraquat could not be explained by a general fitness difference as all strains exhibited comparable growth in the absence of paraquat. Of note, expressing *L. biflexa sodB* did not significantly affect *L. interrogans* virulence (Fig. S3). These findings demonstrate that the presence of a SOD in *L. interrogans* does not improve the fitness in the presence of superoxide unless a cytoplasmic H_2_O_2_ detoxification system is provided. This supports a scenario whereby peroxidases and/or catalases have evolved in pathogenic *Leptospira* spp. accordingly with the loss of a cytoplasmic SOD.

### Long-lasting adaptation of *L. interrogans* to superoxide stress

We tested whether pathogenic *Leptospira* spp., despite the absence of a SOSE, have evolved alternative defense mechanisms and could adapt to superoxide. As previously reported (13–15), when WT *L. interrogans* are cultivated in the presence of paraquat, bacterial growth resumes after a ~10 day-lag phase (Fig. S4, cycle 1). Exponentially growing *L. interrogans* in the presence of paraquat were used to inoculate fresh EMJH with and without paraquat (Fig. S4, cycle 2). This paraquat-selected population exhibited a 3-fold reduced lag phase (~3.5 days) in the presence of paraquat compared with a strain not previously cultivated in the presence of paraquat (Fig. S4, compare cycles 1 and 2).

**Fig 3:**
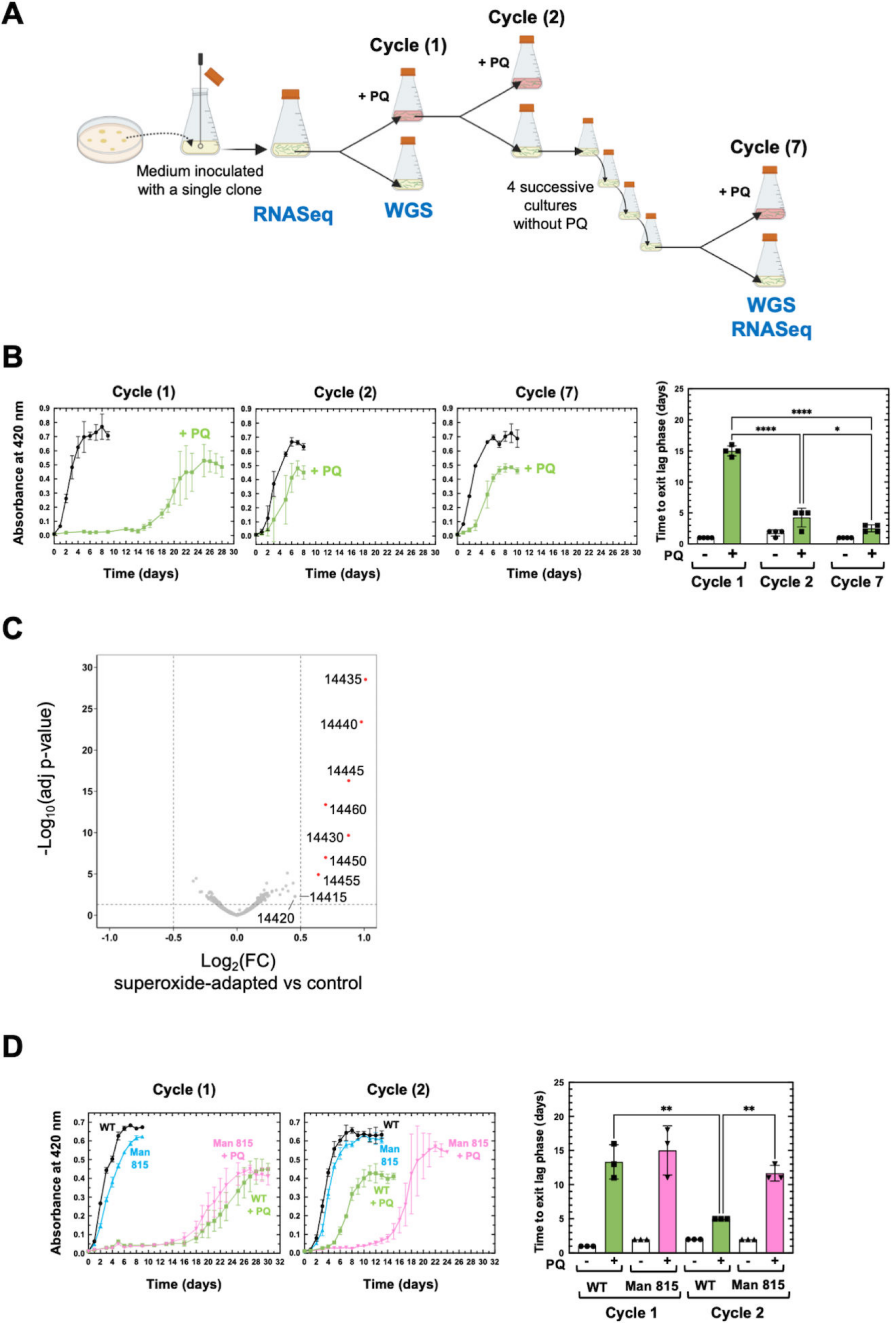
Adaptation of pathogenic *Leptospira* spp. to superoxide. (**A**) Schematic representation of the pipeline for experimental adaptation of *L. interrogans* to superoxide. The adaptation was performed by cultivating monoclonal *L. interrogans* in the absence or presence of 2 µM paraquat (PQ) until exponential phase (cycle 1). Bacteria grown in the presence of paraquat were used to inoculate a fresh medium containing 2 µM paraquat (cycle 2) or passed several times in a medium in the absence of paraquat. The cycle numbers are the number of *in vitro* passages. The steps where whole genome sequencing (WGS) and RNA-seq had been performed are indicated. (**B**) Growth curve of *L. interrogans* WT strain at different stages of the adaptation in the absence (black circle) or presence (green square) of 2 µM paraquat (PQ). Growth curves were performed by inoculating EMJH medium with exponentially growing *Leptospira* and the growth was assessed by measure of absorbance at 420 nm. Time to exit the lag phase (as defined by the first day where the cultures exhibit an OD ≥0.05) was plotted for each cycle, with white bars (circle) and green bars (square) corresponding to growth in the absence or presence of paraquat, respectively. Data are the mean and SD of four independent experiments. Statistical analysis was done by a one-way ANOVA test with Tukey’s multiple comparisons test (****, *P*-value < 0.0001; *, *P*-value = 0.012). (**C**) Volcano representation of the DEGs in the superoxide-adapted strain. Differential expressions are expressed as Log_2_FC of the adapted strain (at cycle 7) versus the control strain. Significantly upregulated ORFs (cutoffs of Log_2_FC > 0.5, adj. *P*-value < 0.05 indicated by vertical and horizontal dashed lines) are represented in red and labelled with ORF number according to UP-MMC-NIID LP strain (16). Please note that there is no significantly downregulated ORF. (**D**) Growth of the superoxide-adapted WT (black circles and green squares) and Man815 mutant (inactivated in LIMLP_14460, blue and pink triangles) strains at cycles 1 and 2 (obtained as described for panel **A**) in the absence (black circles and blue triangles) or presence of 2.5 µM paraquat (green squares and pink inverted triangles). Time to exit lag phase was plotted at cycles 1 and 2, as described for panel **B**, with white bars and colored bars (green and pink) corresponding to growth in the absence or presence of paraquat, respectively. Circles and squares (white and green bars) are WT samples, and triangles (white and pink bars) are Man815 mutant samples. Data are the mean and SD of three independent experiments. Statistical analysis was done by a one-way ANOVA test with Tukey’s multiple comparisons test (**, *P*-value = 0.0011 and *P*-value = 0.0093).

To characterize the bacterial population selected under this condition, adaptation was repeated using a clonal population. A single clone (clone 3) was used to inoculate EMJH medium and two consecutive exposures to paraquat were performed as described above (Fig. 3A, cycles 1 and 2). The bacterial population selected in the presence of paraquat at cycle 1 exhibited, as before, a reduced lag phase when cultivated in the presence of paraquat (Fig. 3B, cycle 2). When this culture was propagated for five consecutive passages in the absence of paraquat, the bacterial population still exhibited a reduced lag phase (~2.5 days) when cultivated in the presence of paraquat (Fig. 3B, cycle 7). We further propagated the paraquat-adapted population and the original culture for 39 consecutive passages (cycle 46, ≈94 generations) in the absence of paraquat. We observed that the paraquat-adapted population still conserved its adaptive phenotype in the presence of paraquat (Fig. S4C and D, cycle 46). The paraquat-adapted population neither exhibited any fitness defect when compared with the non-adapted original population grown in EMJH in the absence of paraquat nor did it exhibit a higher fitness in the presence of H_2_O_2_ (Fig. 3B; Fig. S4E). Altogether, these findings demonstrate that *L. interrogans* can acquire a specific long-lasting adaptation to superoxide at no fitness cost in the absence of a SOSE.

To identify the mechanisms underlying this adaptation, the genome of the superoxide-adapted population at cycle 7 (Fig. 3A) was sequenced and compared with that of the original non-adapted population (clone 3) and of the non-adapted population at cycle 1. Short-read Illumina sequencing showed the presence of 1 single nucleotide polymorphisms (SNP) and five indels in all samples that were already present in the original clone 3 (Table S3). Long-read Nanopore sequencing detected a much higher number of variations. Many SNPs and indels were located in repetitive nucleotide stretches and were probably sequencing artifacts. In addition, many were not detected in all replicates, indicating heterogeneity between samples. Indeed, 6–64 variations were identified, respectively, in each of the four replicates of the adapted strain that were not present in the original clone 3. However, none of them were present in all of the four replicates (Table S3). Therefore, no SNPs, indel, or genome rearrangements could explain the adaptation phenotype.

Comparative RNA-seq analysis indicated that only 11 genes were upregulated in the paraquat-adapted strain (0.4<Log_2_FC < 1, *P-*adj <0.02) (Table S4). These genes encode poorly characterized proteins of the same locus (LIMLP_14415-14460), including an AsrR transcriptional regulator, a DoxX-like protein (LIMLP_14420), three START domain-containing proteins (LIMLP_14430–14440), a DHFR domain-containing protein (LIMLP_14455), and an MFS transporter (LIMLP_14460) (Fig. 3C; Fig. S5). In order to determine the role of this gene cluster in the adaptation to superoxide, we assessed the ability of a mutant inactivated in the LIMLP_14460-encoding MFS transporter to acquire a tolerance to paraquat. Inactivation of LIMLP_14460 did not impair the growth of *L. interrogans* in the presence of paraquat (Fig. 3D, cycle 1, Man815 mutant compared with the WT strain). However, when the exponentially growing, Man815 mutant strain isolated in the presence of paraquat (cycle 1) was cultivated a second time in the presence of paraquat (cycle 2), the lag phase was not significantly reduced as observed for the WT (Fig. 3D, cycle 2). This shows that the MFS transporter encoded by LIMLP_14460 participates in the adaptation to superoxide. Altogether, these findings indicate that the emergence of adaptation to superoxide in *L. interrogans* is probably independent of permanent genome modifications and is partly due to inheritable increased expression of the LIMLP_14415–14460 gene cluster.

### The transcriptomic and proteomic responses to superoxide suggest a metabolism reprogramming

**Fig 4:**
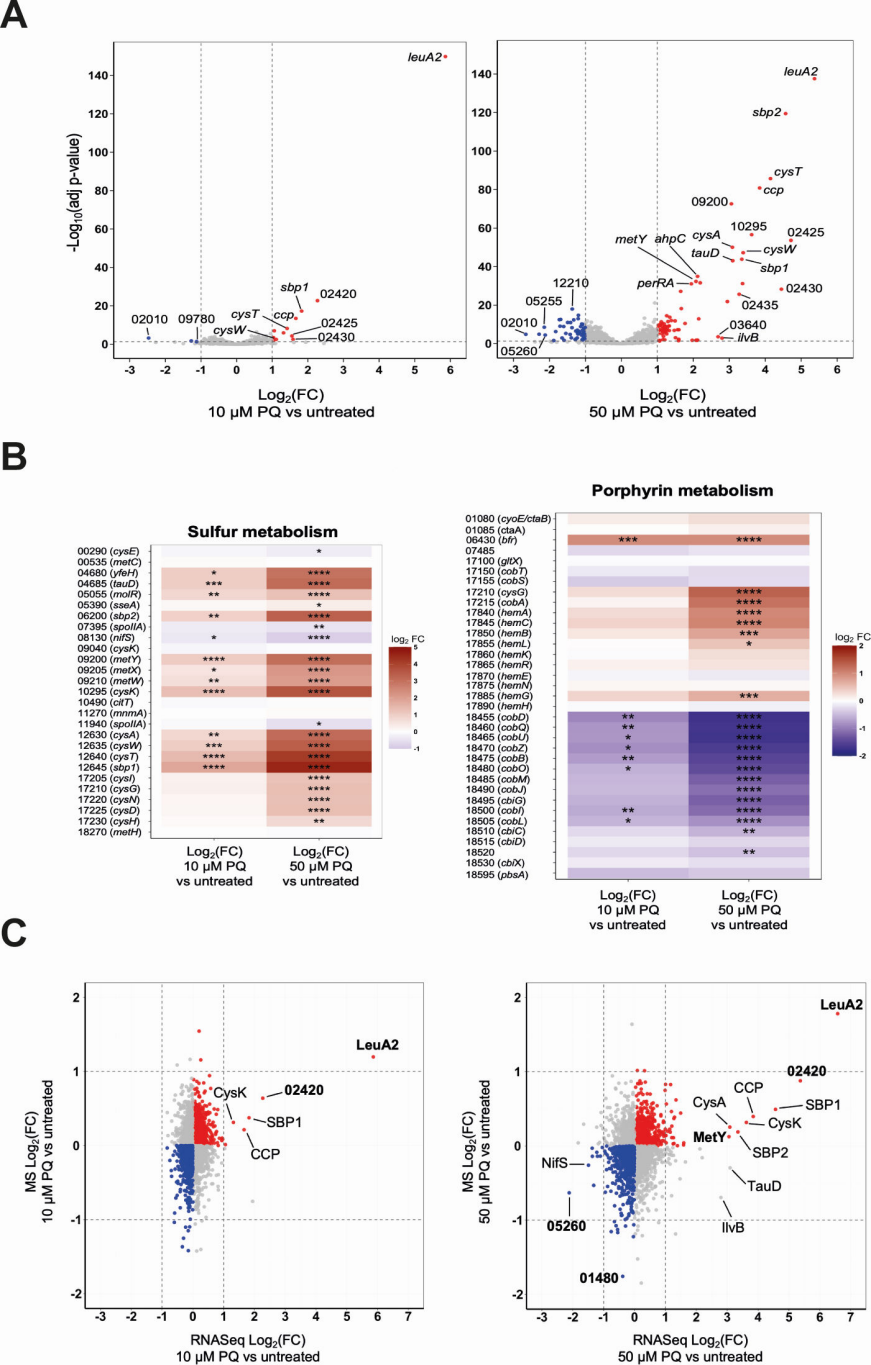
Transcriptomic and proteomic response of *L. interrogans* to superoxide. (**A**) Volcano representation of the DEGs in the presence of 10 (left panel) or 50 µM (right panel) paraquat (PQ). Differential expressions are expressed as Log_2_FC of PQ-treated versus untreated strain. Significantly downregulated and upregulated ORFs (cutoffs of |Log2FC| > 1, adj. *P*-value < 0.05 indicated by vertical and horizontal dashed lines) are represented in blue and red, respectively. Selected genes are labeled by gene name or ORF number according to UP-MMC-NIID LP strain (16). (**B**) Heat map of DEGs related to sulfur (left panel) or porphyrin (right panel) metabolism in the presence of 10 or 50 µM PQ. Gene names and numbers (according to UP-MMC-NIID LP strain) are indicated on the left. The heat map color from blue to red indicates low to high Log_2_FC. Statistical analyses were performed as described in Materials and Methods (*: adj. *P*-value < 0.05, **: adj. *P*-value < 0.01, ***: adj. *P*-value < 0.001, ****: adj. *P*-value < 0.0001). (**C**) Correlation between DEGs (obtained by RNA-seq) and differentially produced proteins (obtained by mass spectrometry [MS]) in the presence of 10 (left panel) or 50 µM (right panel) PQ. Blue and red dots indicate down- and up-regulated ORFs, respectively. Factors that are significantly differentially expressed in both techniques are highlighted in bold.

To identify superoxide-induced mechanisms in pathogenic *Leptospira*, RNA-seq and mass spectrometry analyses were performed on *L. interrogans* exposed to 10 and 50 µM of paraquat. These conditions were chosen, as they do not lead to more than 22% of bacterial death (Fig. S6). 18 and 139 ORFs were differentially expressed in the presence of 10 and 50 µM of paraquat, respectively (|Log_2_FC| > 1, *P-*adj <0.05) (Fig. 4A; Tables S5 to S7), showing a dose-dependent effect.

Several genes encoding oxidative stress-related factors were upregulated in the presence of paraquat. Notably, genes encoding the catalase KatE, the peroxiredoxin AhpC1, the cytochrome C peroxidase CcpA, as well as the two peroxide stress regulators PerRA and PerRB, exhibited an increased expression upon exposure to paraquat (Table S6). This indicates that paraquat triggers oxidative stress in *Leptospira*.

Importantly, differentially expressed genes (DEGs) were enriched in metabolic pathways (Fig. S6). Remarkably, the most upregulated gene at both concentrations encodes a 2-isopropylmalate synthase (*leuA2*), an enzyme that catalyzes the first step of leucine biosynthesis pathway (Fig. 4A; Fig. S7).

In addition, many genes related to the import and metabolism of sulfur-containing molecules were significantly upregulated in the presence of paraquat (Fig. 4A and B; Fig. S7; Table S6). Genes encoding components of an ABC sulfate import complex (*sbp1, sbp2*, and *cysAWT*) and enzymes of the assimilatory sulfate reduction that catalyze the conversion of sulfate (SO_4_^2-^) to sulfide (H_2_S) (i.e., *cysDNHI*), all exhibited increased expression in the presence of paraquat. Genes of the siroheme biosynthesis pathway (*cysG* and *cobA,* encoding a siroheme synthase and uroporphyrinogen-III C-methyltransferase, respectively) were as well upregulated by paraquat. Siroheme is an essential cofactor of the sulfite reductase CysI, necessary to reduce sulfite into sulfide. Another source of sulfite in bacteria is the sulfur-containing amino acid taurine. LIMLP_04680, which encodes a putative bile acid:sodium symporter for taurine uptake (homolog to *Escherichia coli yfeH*), and the adjacent gene *tauD*, which encodes a putative taurine dioxygenase catalyzing the release of sulfite from taurine, were upregulated upon exposure to paraquat (Fig. 4B; Fig. S7; Table S6).

The assimilatory sulfate reduction pathway allows the incorporation of sulfide into cysteine and methionine. The reaction of H_2_S and O-acetyl L-serine leads to the synthesis of L-cysteine and is catalyzed by the cysteine synthase CysK. The reaction of H_2_S with O-acetyl L-homoserine, which produces L-homocysteine, is catalyzed by a homocysteine synthase MetY. Both *cysK* and *metY* exhibited increased expression in the presence of paraquat (Fig. 4B; Fig. S7; Table S6). However, the gene encoding the methionine synthase MetH that converts homocysteine into methionine is not differentially expressed in the presence of paraquat (Table S5).

Nearly 50% of down-regulated genes encoded proteins with unknown function (Table S7). Other encoded factors of different metabolic pathways, including ATP (*atpGDC*) and cobalamin (*cobDQUZBOMJIL*, *cbiGCD*) biosynthesis (Fig. 4B; Table S7).

Analysis of the proteome in the same conditions confirmed the significant upregulation of LeuA2, LIMLP_02420-encoded protein and the downregulation of MauG (encoded by LIMLP_05260) (Fig. 4C; Table S8). In addition, several factors of the sulfur assimilatory pathway (Sbp1, Sbp2, CysA, CysN, CysD, CysH, CysK, and MetY) had an increased cellular content, although with a Log_2_FC lower than 1 (Fig. 4C; Table S8).

Altogether, these findings indicate that in the absence of SOSEs, the paraquat-induced oxidative stress triggers a metabolic reprogramming toward leucine and sulfur-containing amino acids (Cys and Met) biosynthesis pathways.

### The superoxide and peroxide-induced transcriptional responses are partially distinct

Superoxide exposure triggers the upregulation of several genes of the PerRA and H_2_O_2_ regulons, that is, *katE*, *ahpC1*, and *ccpA*, probably because its spontaneous reduction leads to H_2_O_2_ production. To determine to what extent the superoxide and peroxide transcriptional responses overlap, we compared DEGs in the presence of paraquat and H_2_O_2_. We previously determined that 505 ORFs of *L. interrogans* are significantly differentially expressed in the presence of 1 mM H_2_O_2_ (14) (Table S9); 84% (*N* = 422) of the H_2_O_2_ stimulon is not shared with the superoxide stimulon, and 40% (*N* = 83) of the 137 DEGs upon exposure to 50 µM paraquat are not regulated by H_2_O_2_. *KatE*, *ahpC1*, *ccpA*, *perRA*, *perRB*, *clpB*, and the heme and cobalamin biosynthesis genes were deregulated with both ROS (Table S9). Therefore, a subset of the upregulation observed upon exposure to superoxide is probably due to the presence of H_2_O_2_ produced from the reduction of superoxide. However, several genes of sulfate assimilatory pathway (including *sbp1*, *sbp2*, *cysAGIKNTW*, *yfeH*, *tauD*, and *metWXY*), *leuA2,* and the LIMLP_02420-02435 cluster were not or less upregulated in the presence of H_2_O_2_ than upon exposure to paraquat, suggesting that their upregulation is mostly due to specific superoxide-mediated stresses.

### Distinct differential expression of the *leuA* paralogs in the presence of superoxide

**Fig 5:**
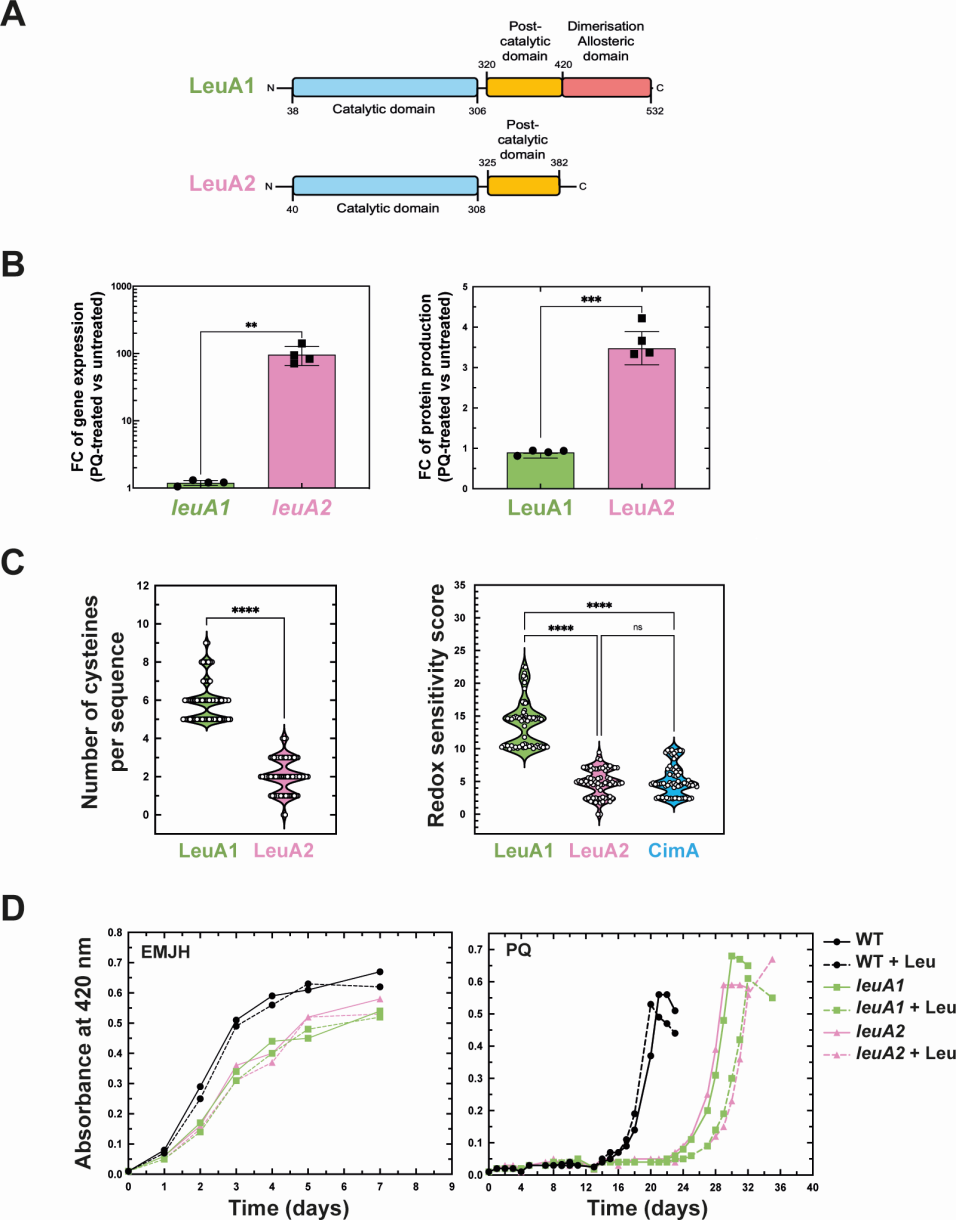
Role of LeuA1 and LeuA2 in the *L. interrogans* fitness in the presence of superoxide. (**A**) Schematic representation of the domain organization of LeuA1 (LIMLP_08570) and LeuA2 (LIMLP_15720) 2-isopropylmalate synthases with the catalytic pyruvate carboxyltransferase HMGL-like domain in blue, post-HMGL-like domain in yellow, and dimerization allosteric domain in red. (**B**) Gene (left panel) and protein (right panel) expression of LeuA1 (green bar, circle) and LeuA2 (pink bar, square) upon exposure to superoxide. Differential expressions are expressed as FC (50 µM PQ-treated versus untreated). Data are mean and SD of four independent experiments. Statistical analysis was done by a paired t test (**: *P*-value < 0.05, ***: *P*-value = 0.0009). (**C**) Number of cysteine residues (left panel) and predicted redox-sensitive score (as analyzed by IUPRED2A-redox [19]) (right panel) of LeuA1 (LIMLP_08570, 532 AA) (in green), LeuA2 (LIMLP_15720, 428 AA) (in pink), and CimA (LIMLP_07785, 516 AA) (in blue) in 68 *Leptospira* species. Each symbol represents a species. Statistical analysis was done by a one-way ANOVA test with Tukey’s multiple comparisons test (****: *P*-value < 0.0001). (**D**) Growth of *L. interrogans* WT (black circle), *leuA1* (green square), and *leuA*2 (pink triangle) mutant strains in EMJH medium (left panel) or in the presence of 3 µM paraquat (PQ, right panel) with (dashed lines) or without (solid line) 2 mM leucine. Growth was assessed by measure of absorbance at 420 nm. Data are one replicate representative of 3 three independent biological replicates.

*LeuA2* (LIMLP_15720) was the most upregulated gene upon exposure to paraquat. Unlike most model bacteria, the *L. interrogans* genome possesses two *leuA* paralogs, i.e., *leuA1* (LIMLP_08570) and *leuA2* (LIMLP_15720). Both ORFs exhibit the canonical catalytic domains of a 2-isopropylmalate synthase; however, LeuA2 lacks the C-terminal domain responsible for an allosteric inhibition by leucine (Fig. 5A; Fig. S8) (17). Unlike LeuA2, LeuA1 was not differentially expressed in the presence of paraquat (Fig. 5B). Genus-wide comparative analysis revealed that LeuA1 and LeuA2 homologs contain 6 and 2 cysteine residues on average, respectively (Fig. 5C). Interestingly, LeuA1 was predicted to be three times more redox-sensitive than LeuA2 (Fig. 5C). Similar analysis on a citramalate synthase (CimA) that catalyzes the first reaction of the isoleucine biosynthesis pathway in *Leptospira* (18) and whose length is close to that of LeuA1 supported that the determination of the redox sensitivity score is independent of protein length. In addition, despite leucine being the most frequent amino acid in *L. interrogans*, its abundance was not affected by the paraquat treatment (Fig. S8 and S9). Also, LeuA2 is the only factor of the leucine biosynthesis pathway that was upregulated by superoxide (Fig. S7).

To determine whether LeuA1 and LeuA2 could have a role in tolerance to superoxide, the growth of WT, *leuA1,* and *leuA2* mutant strains were assessed in the presence of paraquat. Inactivating *leuA1* or *leuA2* slightly impaired the growth of *L. interrogans* in the EMJH medium and severely compromised growth in the presence of paraquat (Fig. 5D). Addition of leucine did not improve the fitness of *L. interrogans* WT and mutant strains in the presence or absence of paraquat (Fig. 5D).

### Superoxide exposure leads to an increase in cysteine oxidation

**Fig 6:**
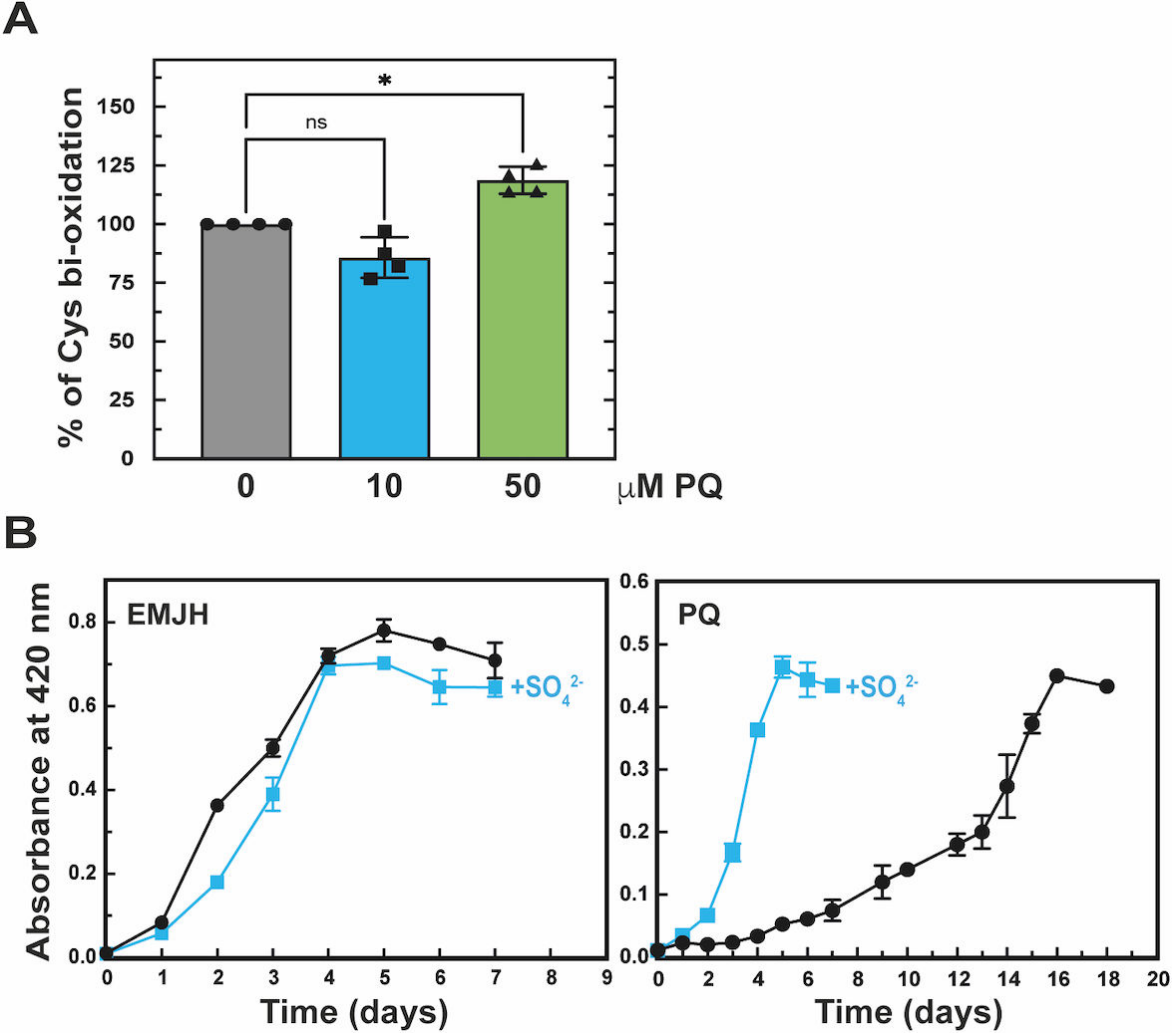
Addition of sulfate increases *L. interrogans* fitness in the presence of superoxide. (**A**) Mass spectrometry-based quantification of cysteine bi-oxidation (sulfinic acid) upon exposure to 0 (gray bar, circle), 10 (blue bar, square), or 50 (green bar, triangle) μM paraquat (PQ). Data are mean and SD of four independent experiments. Statistical analysis was done with a one-way ANOVA repeated measures with Dunnett’s multiple comparisons test (*, *P*-value = 0.0123). (**B**) Growth of *L. interrogans* WT strain in EMJH medium in the absence (left panel) or presence of 2 µM paraquat (PQ) (right panel) with (blue line) or without (black line) 20 mM Na_2_SO_4_. Growth was assessed by measure of absorbance at 420 nm. Data are mean and SD of three independent experiments.

To assess the amino acid oxidation triggered by superoxide, proteinogenic amino acids detected in the proteome were quantified in *L. interrogans* upon exposure to paraquat. We determined that cysteine is the least abundant AA in the *L. interrogans* proteome (both *in silico* and by mass spectrometry analysis), and exposure to paraquat did not affect its abundance (Fig. S9 and S10). However, exposure to 50 µM paraquat led to a significant increase in cysteine bi-oxidation (sulfinic acid) (Fig. 6A; Fig. S10).

Determining the proteome-wide oxidation ratios demonstrated that only a subset of proteins was oxidized in cysteine. DnaG primase (encoded by LIMLP_08540) was the protein exhibiting the highest significant increase in its cysteine sulfinic acid content (at Cys321) upon exposure to paraquat (Fig. S11; Table S8). Interestingly, DnaG oxidation correlated with a replication arrest when *L. interrogans* was exposed to paraquat (Fig. S11). Overall, downregulated genes and proteins were enriched in cysteine-containing proteins (Fig. S11), and almost none of the predicted Fe-S cluster-containing proteins exhibited increased expression upon exposure to paraquat (Fig. S11). In addition, exposure to paraquat did not significantly affect aconitase activity, used here as a proxy for assessing damage to iron-sulfur cluster-containing proteins (Fig. S11). Overall, this demonstrates that, in the conditions tested here, superoxide exposure causes cysteine oxidation without leading to Fe-S cluster inactivation.

### Sulfate increases adaptation to superoxide

The upregulation of several factors of the sulfate assimilatory pathway upon exposure to superoxide strongly suggests a role of this pathway in the adaptation to superoxide. To confirm this, metabolites (sulfate, sulfite, and sulfide) and the final product (cysteine) of the pathway were added to the culture medium. Although the growth of *L. interrogans* in the presence of paraquat was not improved when sulfite (SO_3_^2-^), sulfide donors (Na_2_S, NaSH), cysteine, or cystine (cysteine dimer) were added to the medium, the addition of sulfate did increase the ability of *L. interrogans* to grow in the presence of paraquat (Fig. 6B; Fig. S12). Exposure of *L. interrogans* to paraquat did not trigger an increase in sulfide (H_2_S) production (Fig. S12), indicating that H_2_S is not the final product of the sulfur assimilatory pathway upon paraquat exposure. Thus, we confirmed that sulfate assimilation improves the adaptation of *L. interrogans* to superoxide.

## DISCUSSION

In the present study, we demonstrate that all P clade *Leptospira* species lack SOD. No superoxide removal activity could be observed upon exposure to superoxide (Fig. S3), confirming that the life-threatening pathogen *L. interrogans* is devoid of an alternative superoxide detoxification machinery. Importantly, we reveal that the SOD present in the LLCA was maintained in saprophytes but specifically lost by pathogens. Our findings indicate that SOD loss could have occurred stepwise, as observed within the S2 clade, leading to P species devoid of any *sod*. In addition, maintaining a SOD activity in *Leptospira* species requires the concomitant presence of cytoplasmic H_2_O_2_ detoxification enzymes, such as KatG. What could have driven SOD loss? Since the main function of this enzyme is to rapidly eliminate the toxic superoxide produced during aerobic metabolism, it is tempting to propose that the aerobic metabolism of P-clade *Leptospira* species is reduced in the low oxygenated environment encountered within host tissues. Alternatively, SOD loss could have been driven by a reduced host-produced superoxide accumulation at the site of colonization during infection.

The absence of a SOSE in bacteria is rare (estimated at 9–13%), as demonstrated here and in a recent study (20). The loss of a SOD is unforeseen in pathogenic bacteria, as this enzyme has proven to be necessary for the virulence of a variety of bacteria (21–23), and some pathogens have even acquired additional extracellular SOD to fight host-derived superoxide (24, 25). Furthermore, our finding that SOSE-deficient organisms are equally found among aerobes and anaerobes suggests that aerobicity does not determine the prevalence of SOSEs. Overall, this challenges the universality of the oxygen toxicity theory and the essentiality of superoxide scavenging activity for aerobes.

Despite the absence of any SOSE, we successfully selected a long-lasting superoxide-adapted population after a unique exposure to superoxide. This adaptation is inherited for at least 94 generations at no fitness cost. When the adapted population was analyzed for the presence of any mutation that could have been acquired during the adaptation, no SNPs nor Indels were detected in a reproducible manner in all the replicates. The possibility that heterogeneity of the variations present in different replicates of the adapted population could have led to the same phenotype, that is, greater tolerance to paraquat, cannot be totally excluded. However, such heterogeneity was seen only with long-read sequencing and not reproduced by short-read sequencing technology, making it very likely that this heterogeneity represents sequencing artifact. Therefore, adaptation to paraquat could not be explained by the acquisition of any mutation.

An upregulated gene cluster was identified in the superoxide-adapted strain. Our finding that inactivating one of these genes (LIMLP_14460) impaired the adaptation of *L. interrogans* to superoxide supports a role of this cluster in the ability of *L. interrogans* to adapt to superoxide. LIMLP_14460 encodes an MFS family transporter, typically involved in toxic compound efflux. A recent study of adaptive laboratory evolution in *E. coli* showed that adaptation to paraquat was also associated with mutations in transporter-encoding genes (26). Confirming the role of the LIMLP_14460-encoded MFS transporter in tolerance to paraquat would require complementing the mutant inactivated in LIMLP_14460. Nevertheless, we propose that modulating the import/export of redox-cycling compounds such as paraquat is probably a common mechanism for adapting to these ROS-producing molecules and may be the most efficient strategy for preventing superoxide accumulation in SOSE-deficient organisms. Notably, our findings reveal as well that this phenotypic trait might be independent of any permanent genetic modification and may rely instead on inheritable increased gene expression. Epigenetic modifications could be responsible for triggering such increases in gene expression. Interestingly, the adaptation to paraquat was characterized by a reduced lag phase rather than a decrease in the doubling time, suggesting that the superoxide-adapted population is more efficiently prepared for the transcriptional and metabolic reprogramming necessary to exit the lag phase. In a previous study, we had identified a PerR regulator in *L. interrogans* (PerRB) whose inactivation led to increased fitness in the presence of superoxide (15), corroborating that pathogenic *Leptospira* species possess alternative transcriptionally controlled defense mechanisms to compensate for the loss of SOD.

In this study, we demonstrate that the PerRA regulon (including *katE*, *ahpC1*, and *ccpA* genes) is derepressed when *L. interrogans* are exposed to paraquat, indicating that exposure to this compound led to the production of H_2_O_2_ (and consequently of hydroxyl radical, ^•^OH). Considering the absence of any SOSE in these bacteria, H_2_O_2_ production could result from spontaneous reduction of superoxide or be catalyzed by a yet unknown enzyme with superoxide reduction activity. We could not identify any factor with such activity with the transcriptomic/proteomic approach in this study. Moreover, total extracts of *L. interrogans* failed to significantly eliminate superoxide, even in the presence of paraquat (Fig. S3D). This strongly suggests that pathogenic *Leptospira* are devoid of any enzymatic activity that rapidly scavenges superoxide. Identification of molecules that non-enzymatically promote superoxide reduction in *L. interrogans*, if any, will require additional studies.

**Fig 7:**
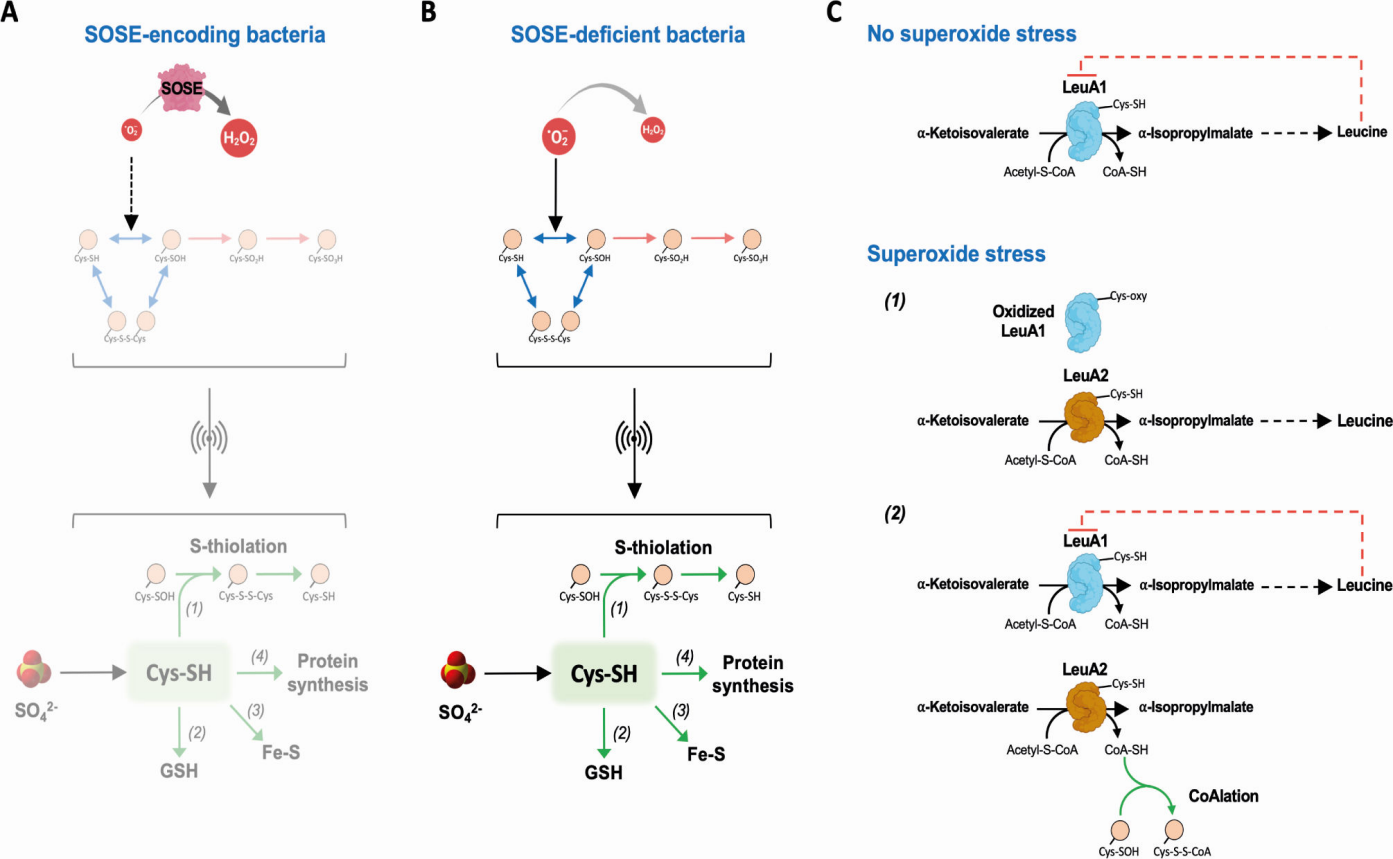
Model for the superoxide stress response in SOD-deficient bacteria. (**A**) In SOSE-containing organisms, rapid reduction of superoxide into hydrogen peroxide prevents cysteine oxidation and upregulation of the sulfur assimilatory pathway. (**B**) In SOSE-deficient organisms, such as *L. interrogans*, the low rate of superoxide reduction favors reversible (disulfide bond and sulfenic acid) and irreversible (sulfinic and sulfonic acids) cysteine oxidation, leading to protein denaturation and damage. The upregulation of the sulfur assimilatory pathway might result in an increased cysteine cellular content. Cysteine could contribute to resistance to oxidative damage by protecting thiol groups of cysteine residues from oxidation through thiolation (*arrow 1*), by being used for glutathione (GSH) synthesis (*arrow 2*), by providing sulfur for iron-sulfur clusters (*arrow 3*), by being incorporated in newly synthesized proteins to replace irreversibly damaged protein during oxidation (*arrow 4*). Accumulation of oxidized cysteines might signal the upregulation of the sulfur assimilatory pathway. (**C**) Model for the role of LeuA2 in the superoxide stress response. In normal growth conditions, LeuA1 is the isopropylmalate synthase that catalyzes the first step of leucine synthesis pathway (represented on the top). The inhibition of LeuA1 by leucine is represented by a dashed red line. Two mechanisms can explain LeuA2 upregulation upon superoxide stress. In the first mechanism, LeuA1 is oxidized and LeuA2 production compensates for the inactivation of LeuA1 (mechanism 1). In another mechanism, LeuA2 is produced during oxidative stress to trigger the accumulation of CoASH that protects oxidized cysteine by CoAlation (mechanism 2). In the latter case, LeuA1 could be among the CoAlated proteins. LeuA2 lacks the C-terminal regulatory domain and is not inhibited by leucine.

In the present study, the sulfate assimilatory reduction pathway was strongly upregulated upon exposure to superoxide. The importance of this pathway is supported by our observation that sulfate improves the fitness of *L. interrogans* in the presence of superoxide. This pathway is also upregulated in the presence of paraquat in *E. coli* and in *Bacillus subtilis* (27, 28), suggesting that its role in tolerance to superoxide is conserved in bacteria. This pathway leads to the production of H_2_S, a molecule with antioxidant properties (29, 30), and sulfur-containing amino acids (Cys and Met). We could not detect H_2_S production in *L. interrogans*, even in the presence of paraquat (Fig. S12). It is therefore unlikely that the upregulation of the sulfate assimilatory reduction pathway leads to the accumulation and protective effect of H_2_S. In addition, the facts that a *metY* mutant did not exhibit any fitness defect with superoxide (Fig. S12) and that *metH* is not deregulated with superoxide make it very unlikely that methionine participates in defense against superoxide. The upregulation of the cysteine synthase CysK strongly suggests that the purpose of the superoxide-triggered sulfate assimilatory reduction pathway is cysteine synthesis. Cysteine could either be used for the synthesis of the redox buffer glutathione or act as thiol protectant through S-thiolation, as demonstrated in *Staphylococcus aureus* and *B. subtilis* (31, 32) (Fig. 7A and B). Cysteines could also be incorporated in newly synthesized polypeptides to replace damaged proteins containing irreversibly oxidized cysteines (Fig. 7A and B). Finally, cysteine catabolism could contribute to iron-sulfur cluster assembly (Fig. 7A and B). However, no genes involved in iron-sulfur cluster assembly, glutathione biosynthesis, or cysteine catabolism were upregulated in our conditions. Although the fate of cysteine upon exposure to superoxide is yet to be determined, we demonstrate that the sulfate assimilatory reduction pathway plays a central role in adaptation to superoxide. Consistent with this, CysK is among the four most abundant proteins in *L. interrogans* (33), thereby suggesting that pathogenic *Leptospira* compensated for the absence of SOSEs by maintaining high levels of cysteine production.

The most upregulated factor upon exposure to superoxide was the 2-isopropylmalate synthase that catalyzes the first step of leucine biosynthesis encoded by *leuA2*. Of the two *leuA* paralogs present in *Leptospira* spp., only *leuA2* was upregulated by superoxide. In the absence of oxidants, LeuA1 is 3.7 times more abundant than LeuA2 (33), and LeuA2 does not contain the C-terminal domain responsible for allosteric inhibition by leucine (17). It is very likely that LeuA1 is the enzyme in charge of maintaining leucine homeostasis in the absence of oxidants, whereas the specific upregulation of LeuA2 by superoxide would allow to compensate for the oxidative damage to LeuA1 (Fig. 7C, mechanism 1). This hypothesis is supported by the fact that LeuA1 homologs, which contain more cysteine than LeuA2s, are predicted to be more redox-sensitive. Another hypothesis is that the conversion of 2-keto-isovalerate and acetyl-CoA to 2-isopropylmalate, when catalyzed by LeuA2, would lead to coenzyme A (CoA-SH) accumulation, allowing protection of cysteine thiols from oxidation by CoAlation (34, 35) (Fig. 7C, mechanism 2).

A classical interpretation of the role of leucine and cysteine in superoxide stress stems from the conditional auxotrophy of *E. coli sod* mutants for several amino acids under aerobic conditions (36, 37). This was explained by the inactivation of Fe-S cluster of enzymes involved in amino acids biosynthesis (38, 39). In our conditions, the addition of cysteine or leucine did not improve the fitness of *L. interrogans* in the presence of superoxide, and aconitase activity was not altered by exposure to superoxide. Although we cannot exclude that higher paraquat doses would lead to Fe-S cluster inactivation in *L. interrogans*, we speculate that SOSE-deficient organisms, such as pathogenic *Leptospira* spp., have evolved higher Fe-S cluster resistance from oxidative damage.

To conclude, by exploring the adaptation of a naturally SOSE-deficient species, like pathogenic *Leptospira*, to superoxide, this study offers a revisited perspective on our understanding of the classical dogma of oxygen toxicity. We demonstrate that these aerobes are capable of a SOSE-independent adaptation to superoxide and emphasize the importance of metabolism-based strategies to compensate for the absence of an enzymatic superoxide detoxification activity.

## MATERIALS AND METHODS

### Bacterial strains and growth condition

*L. interrogans* serovar Manilae strain L495 and transposon mutant strains (see Table S10 for a complete description of the transposon mutants used in this study) were grown aerobically at 30°C in Ellinghausen-McCullough-Johnson-Harris medium (EMJH, which contains 180 µM FeSO_4_) (40) with shaking at 100 rpm or in EMJH solid agar medium for 1 month until appearance of colonies. Π1 and β2163 *E. coli* strains were cultivated at 37 ˚C in Luria-Bertani medium with shaking at 37 ˚C in the presence of 0.3 mM thymidine or diaminopimelic acid (Sigma-Aldrich), respectively. Spectinomycin was added to the media at 50 µg/mL when needed. When indicated, the superoxide-generating compound paraquat (1,1′-Dimethyl-4,4′-bipyridinium-2,2′,3,3′,5,5′,6,6′-d8 dichloride), H_2_O_2_ (Sigma-Aldrich), sodium sulfate (Na_2_SO_4_), sodium bisulfite (NaHSO_3_), sulfide donors (Na_2_S, NaSH), leucine, cysteine, cystine (diluted in Tris-HCl), or Tris-HCl were added to the medium. In all growth curves, EMJH medium was inoculated with *Leptospira* culture maintained in the logarithmic phase. Bacterial growth was followed by measuring the absorbance at 420 nm.

### Adaptation experiment

L495 clone 3 was isolated by plating a culture of *L. interrogans* serovar Manilae strain L495 on EMJH plates. Exponentially growing *L. interrogans* polyclonal or L495 clone 3 cultures were used to inoculate EMJH medium in the presence or absence of paraquat (2.0–2.5 μM, as indicated). The bacterial population that reached an exponential phase in the presence of paraquat (cycle 1) was used to inoculate fresh medium containing or not paraquat (cycle 2). Subsequent cycles were obtained by passaging *Leptospira* several times in the absence of paraquat. The number of generations was estimated under the assumption that the average generation time of *L. interrogans* is 20 h and that each passage corresponds to 48 h of exponential growth. Whole genome sequencing was performed on L495 clone 3 at cycles 1 and 7 (see Supplementary Information for detailed procedures), and RNA-seq was performed on L495 clone 3 at the beginning of the experiment and at cycle 7 on *Leptospira* harvested at exponential phase in the absence of paraquat.

### Expression of *L. biflexa sodB* and *katG* in *L. interrogans*

Heterologous expression of *sodB* in *L. interrogans* was performed by PCR amplification of LEPBIa0027 (*sodB*) from genomic DNA of *L. biflexa* serovar Patoc strain Patoc 1 using the primers sodB_F and sodB_R (Table S11). The PCR product was cloned into the pMaGRO vector at NdeI/XbaI restriction sites (41). The resulting plasmid (pMAGRO::*sodB*) was named pSGH1. For heterologous expression of *katG*, a synthetic construct fusing the promoter of *lipL32* until its transcription start site to *katG* (LEPBIa2495) and flanked by BamHI restriction sites was obtained through synthesis (GeneArt, ThermoFisher). Cloning of the fusion was performed by restriction-ligation with BamHI in both empty pMaORI (42) and pMaGRO::*sodB* (pSGH1). The resulting pMaORI::*katG* and pMaGRO::*sodB-katG* plasmids were named pSGH11 and pSGH10, respectively. All plasmids were verified by whole plasmid sequencing with Nanopore long-read technology (Plasmidsaurus Inc, Arcadia, California) and were introduced in *L. interrogans* by conjugation, as previously described (43). Conjugants were selected on solid EMJH agar containing spectinomycin (50 µg/mL) and correct plasmid acquisition was further verified by PCR amplification using the primers pMaORI-A/Mao2 and maoEco1/maoEco2 (Table S11). The WT *L. interrogans* containing empty pMaORI as control was obtained from previous studies (13).

### RNA purification

Virulent *L. interrogans* serovar Manilae strain L495 with less than three *in vitro* passages was used in this study. Four independent biological replicates of exponentially grown WT *L. interrogans* strain were incubated in the presence or absence of 10 µM and 50 µM paraquat for 60 min at 30°C. Each sample was divided in two. One part was used for RNA purification, and the other part was used for mass spectrometry analysis (see below). For the analysis of the superoxide-adapted strain, three independent biological replicates of control (non-adapted) or adapted strains were used. Harvested bacteria were resuspended in 1 mL TRIzol (ThermoFisher Scientific) and stored at −80°C. Nucleic Acids were extracted with chloroform and precipitated with isopropanol as described elsewhere (44). Contaminating genomic DNA was removed by DNAse treatment using the RNAse-free Turbo DNA-free turbo kit (ThermoFisher Scientific) as described by the manufacturer. The integrity of RNAs (RIN >7) was verified by the Agilent Bioanalyzer RNA NanoChips (Agilent technologies, Wilmington, DE).

### RNA sequencing

Libraries were built using the Illumina Stranded Total RNA Prep with Ribo-Zero library kit (Illumina, USA) following the manufacturer’s protocol. Quality control was performed on an Agilent BioAnalyzer. Sequencing was performed on Illumina’s platform NextSeq500 to obtain 70 base single-end reads. The RNA-seq analysis was performed with Sequana 0.16.3 (45). We used the RNA-seq pipeline 0.19.1 (https://github.com/sequana/sequana_rnaseq) built on top of Snakemake 7.32.4 (46). Briefly, reads were trimmed from potential adapters using Fastp 0.23.2 (47) and then mapped to the *L. interrogans* serovar Manilae strain UP-MMC-NIID LP assembly (16) (GCF_001047635.1_ASM104763v1) using Bowtie 2.4.5 (48). FeatureCounts 2.0.1 (49) was used to produce the count matrix, assigning reads to features using the corresponding genome annotation with strand-specificity information. Quality control statistics were summarized using MultiQC 1.17. Statistical analysis on the count matrix was performed to identify differentially regulated genes. Clustering of transcriptomic profiles was assessed using principal component analysis (PCA). Differential expression testing was conducted using DESeq2 library 1.34.0 (50), indicating the significance (Benjamini-Hochberg adjusted p-values, false discovery rate FDR < 0.05) and the effect size (fold-change) for each comparison. Differential expressions were expressed as logarithms to base 2 of fold change (Log_2_FC).

### *In silico* identification of SOD and SOR in bacterial genome databases

The SOR protein sequence from *Pyrococcus furiosus* (UniProt P82385) and the SOD protein sequence from *L. biflexa* (UniProt Q1EMH7) were used as queries for a search with BLAST v2.13.0 (51) against a database constructed using the 1110 established bacterial pathogens infecting humans database (9). Significant hits with e-value cutoff of 0.01 were extracted and aligned with MAFFT v7.467 (L-INS-i algorithm) (52) to generate an HMM profile subsequently used to scan the databases for missing hits with HMMer v3.3.1 (53) with an e-value cutoff of 0.01. Presence of a SOD or SOR was estimated positive for all proteomes containing hits with an e-value cutoff lower than 0.01 for any of the two searches (BLAST or HMMer). To rule out artifacts due to the lack of representativeness of the genome chosen within the species, all SOD/SOR double-negative species were double-checked by running BLASTp with the same queries against all RefSeq curated genomes available within each TaxID in the NCBI BLASTp website (54). Positive hits for this last search were manually corrected in the final table. Aerobicity for the final list of SOD/SOR double-negative species was determined using literature screening.

### Distribution of *sod*, *katE,* and *katG* in *Leptospira* spp.

The SodB protein (LEPBIa0027), the KatE protein (LIMLP_10145), and the KatG protein (LEPBIa2495) sequences were used as a query for a BLAST v2.13.0 search (51) against the reference database of 68 *Leptospira* species (55, 56). Hits with an e-value ≤0.01 were retained and subsequently aligned by MAFFT v7.467 under the L-INS-i algorithm (52). This alignment was used to compute an HMM profile to search for missing hits in the same database with HMMer v3.3.1 (53). All significant hits (e-value ≤0.01) were retained and aligned with MAFFT v7.467 under the L-INS-i algorithm, and tree inference was performed with IQ-TREE v2.0.6 under the best-fitted model of evolution (57). Putative orthologs were extracted from these phylogenies and plotted against a core-genome tree of *Leptospirales* obtained as described previously (12) using the *ggtree* package for R v4.3.2 (58).

### SOD activity

For determination of SOD activity, 100 mL of exponentially growing *L. interrogans* or the respective *sodB*- and *katG*-encoding strains were harvested and resuspended in 800 µL of PBS and sonicated (3 cycles of 30 s) to prepare total protein extracts. Total protein extracts were then assayed for SOD activity using the Superoxide Dismutase Colorimetric Activity Kit (EIASODC, ThermoFisher) following the manufacturer’s recommendations.

### Redox-sensitivity score

Direct orthologs of LeuA1 (LIMLP_08570) and LeuA2 (LIMLP_15720) were searched as abovementioned and submitted to IUPRED2A with redox state option locally (19). For the 68 *Leptospira* species, the redox sensitivity score (i.e., the context-dependent disordered propensity of a protein sequence due to changes in the redox state of its cysteine residues) was obtained by calculating the difference between the score under the redox-plus and the redox-minus profiles across all residues. Similar analysis was performed on the citramalate synthase synthase CimA (encoded by LIMLP_07785, 516 AA) as a control to ensure that the redox sensitivity score is independent of the protein length.

### Proteomic analyses

#### Sample preparation

*Leptospira* cultures (prepared as described for RNA extraction) were harvested and extensively washed with PBS. Pellets were resuspended in a lysis buffer (50 mM Tris-HCl pH 8.0, 2 mM EDTA, 5 mM DTT), and lysis was performed by sonication; 10 µg of the total extract was loaded on a 12% SDS-PAGE. After 1 cm of migration in the resolving gel, proteins were fixed with acetic acid and ethanol and stained by Colloidal Blue. In-gel protein digestion and extraction of tryptic peptides were performed as previously described (59) with minimal variations. Briefly, 1 cm bands were excised from the SDS-polyacrylamide gel, previous to cysteine reduction and alkylation by incubation with 10 mM dithiothreitol (DTT) and 55 mM iodoacetamide (IAA), respectively. In-gel protein digestion was performed overnight at 37°C with sequencing grade trypsin (Promega) in a protease:protein ratio of 1:50 (wt/wt). Tryptic peptides were extracted from the gel with 60% ACN/0.1% trifluoroacetic acid (TFA) by two incubations of 1 h at 30°C. The digestions were dried under vacuum and peptides were desalted using ZipTips C18 microcolumns (Merck Millipore). Desalted peptides were vacuum-dried and resuspended in 0.1% formic acid (FA).

#### LC-MS/MS analysis

An UltiMate 3000 nanoHPLC system (Thermo Fisher Scientific) coupled to a Q Exactive Plus mass spectrometer (Thermo Fisher Scientific, USA) was used to perform LC-MS/MS analysis. Tryptic peptides were loaded into a precolumn (Acclaim PepMapTM 100, C18, 75 µm × 2 cm, 3 µm particle size) and separated in an Easy-Spray analytical column (PepMapTM RSLC, C18, 75 µm × 50 cm, 2 µm particle size) at 40°C using two mobile phases: 0.1% FA in water (A) and 0.1% FA in acetonitrile (ACN) (B). The separation gradient was from 1% to 35% B over 150 min and from 35% to 99% B over 20 min, at a flow rate of 200 nL/min. For data acquisition, the mass spectrometer was set in a positive mode using a top-12 data-dependent method, with an ion spray voltage of 2.3 kV and a capillary temperature of 250°C. The full MS scans were acquired from 200 to 2,000 m/z with a resolution of 70,000 at 200 m/z, an AGC target value of 1E6, and a maximum ion injection time of 100 ms. The precursors were fragmented in an HCD cell and acquired with a resolution of 17,500 at 200 m/z, an AGC target value of 1E4, and a maximum ion injection time of 50 ms. For fragmentation, normalized collision energy (NCE) was used in steps 25, 30, and 35. Dynamic exclusion time was set to 5 s. Each peptide sample was injected twice as technical replicates.

#### Mass spectrometry data analysis

PatternLab for Proteomics V software (PatternLab) (60) was used to perform peptide spectrum matching and label-free quantitative analysis. MS data were searched against a target reverse database generated with PatternLab, including the *Leptospira interrogans* serovar Manilae strain UP-MMC-NIID-LP proteome (downloaded from NCBI, PRJNA287300, 20/09/2021) and the most common protein contaminants. For peptide identification, m/z precursor tolerance was set at 40 ppm. Methionine oxidation, cysteine dioxidation, cysteine trioxidation, and cysteine carbamidomethylation were defined as variable modifications. A maximum of 2 missed cleavages and 2 variable modifications per peptide were allowed. Search results were filtered by the PatternLab Search Engine Processor (SEPro) algorithm with a maximum FDR value ≤1% at protein level and 10 ppm tolerance for precursor ions. PatternLab’s Venn diagram statistical module was performed according to Patternlab’s Bayesian model to determine peptides and proteins uniquely detected in each biological condition using a probability value less than 0.05 (61, 62). PatternLab’s TFold module was used to relatively quantify peptides and proteins present in both biological conditions by a spectrum count-based label-free quantification method. Peptides or proteins present in at least five biological replicates from a total of eight were considered for TFold analysis. This module uses the Benjamini-Hochberg theoretical estimator to deal with multiple *t*-tests, and it maximizes the number of identifications satisfying a fold change cutoff that varies with the p-values (BH q < 0.05) while restricting false differential proteins mainly due to low abundance (63).

### Statistics and reproducibility

Statistical analyses were performed using GraphPad Prism (version 10.3.0) as indicated in the Figure legends. Unless otherwise stated, data represent the mean and standard deviation of at least three independent biological replicates. All RNA-seq analyses were conducted using the Sequana RNA-seq pipeline (0.19.1) with published singularity/apptainer containers (64), all of which are available on Zenodo via the Damona project (https://damona.readthedocs.io).

## Data Availability

RNASeq data were deposited at Gene Expression Omnibus (GEO), with series GSE278514 (WT strain untreated versus treated with paraquat) and GSE278513 (WT strain adapted to paraquat versus original clone). Whole genome sequencing data (WT strain adapted to paraquat versus original clon) were deposited at Sequence Read Archive (SRA) under identifier PRJNA1169012. Mass spectrometry proteomics data (WT strain untreated versus treated with paraquat) were deposited to the ProteomeXchange Consortium via the PRIDE (https://www.proteomexchange.org/) (65) partner repository with the data set identifier PXD055480.

## SUPPLEMENTAL MATERIAL

Table S1 mbio.00645-25-s0001.xlsx Presence of SOD and SOR across bacterial pathogens.

Table S2 mbio.00645-25-s0002.xlsx List of genomes with accession numbers.

Table S3 mbio.00645-25-s0003.xlsx Mutations in the original L495 clone C3 and in the adapted strain.

Table S4 mbio.00645-25-s0004.xlsx DEGs in the adapted strain.

Table S5 mbio.00645-25-s0005.xlsx Total set of DEGs with paraquat.

Tables S6 and S7 mbio.00645-25-s0006.pdf Up- and downregulated DEGs with paraquat.

Table S8 mbio.00645-25-s0007.xlsx Mass spectrometry analysis of the proteome upon exposure to paraquat.

Table S9 mbio.00645-25-s0008.xlsx DEGs comparing paraquat and H_2_O_2_ exposures.

Tables S10 and S11 mbio.00645-25-s0009.pdf Strains, plasmids, and primers used in this study.

Supplemental material mbio.00645-25-s0010.pdf Figures S1 to S12; supplemental experimental procedures and references.

ASM does not own the copyrights to Supplemental Material that may be linked to, or accessed through, an article. The authors have granted ASM a non-exclusive, world-wide license to publish the Supplemental Material files. Please contact the corresponding author directly for reuse.
