# Supplementary material for: Revisiting oxygen toxicity: evolution and adaptation to superoxide in a SOD-deficient bacterial pathogen
Source: mBio. 2025 Jul 23;16(8):e00645-25. doi: 10.1128/mbio.00645-25 (PMC12345160; doi:10.1128/mbio.00645-25)
Supplement: Tables S6 and S7 — Up- and downregulated DEGs with paraquat. [file mbio.00645-25-s0006.pdf]

Old locus[a]	New locus[b]	Gene	Function	Log_2FC 10 μM PQ[c]	Log_2FC 50 μM PQ[c]	
**Metabolism**						
LIMLP_15720*	LIMLP_RS15680	*leuA2*	2-isopropylmalate synthase	5.861	6.579	Leu biosynthesis
LIMLP_03635	LIMLP_RS03630	*ilvB*	Leu metabolism	1.940	2.798	Ileu biosynthesis
LIMLP_06200	LIMLP_RS06190	*sbp2*	ABC transporter permease	NA	3.348	Sulfur assimilatory
LIMLP_10295	LIMLP_RS10280	*cysK*	Cysteine synthase	1.317	3.622	Sulfur assimilatory
LIMLP_12630	LIMLP_RS12600	*cysA ?*	Sulfate ABC transporter (ATPase component)	NA	3.085	Sulfur assimilatory
LIMLP_12635	LIMLP_RS12605	*cysW*	Sulfate ABC transporter permease	1.028	3.387	Sulfur assimilatory
LIMLP_12640*	LIMLP_RS12610	*cysT*	Sulfate ABC transporter permease	1.417	4.145	Sulfur assimilatory
LIMLP_12645*	LIMLP_RS12615	*sbp1*	Sulfate import, ABC transporter permease	1.825	4.565	Sulfur assimilatory
LIMLP_17205	LIMLP_RS17165	*cysI*	Sulfite reductase	NA	1.249	Sulfur assimilatory
LIMLP_17210	LIMLP_RS17170	*cysG*	Siroheme synthase, precorrin-2 dehydrogenase	NA	1.326	Sulfur assimilatory
LIMLP_17215	LIMLP_RS17175	*cobA*	uroporphyrin-III methyltransferase	NA	1.186	Sulfur assimilatory
LIMLP_17220	LIMLP_RS17180	*cysN*	Sulfate adenylyltransferase	NA	1.296	Sulfur assimilatory
LIMLP_17225	LIMLP_RS17185	*cysD*	Sulfate adenylyltransferase	NA	1.343	Sulfur assimilatory
LIMLP_09200*	LIMLP_RS09185	*metY*	O-acetyl homoserine aminocarboxypropyltransferase	1.058	3.058	Met biosynthesis
LIMLP_09205	LIMLP_RS09190	*metX*	Homoserine acetyltransferase	NA	2.191	Met biosynthesis
LIMLP_09210	LIMLP_RS09195	*metW*	homoserine O-acetyl-transferase	NA	2.075	Met biosynthesis
LIMLP_12540	LIMLP_RS12510		Histidinol-phosphate aminotransferase	NA	1.122	Tyr, His, Phe, Trp biosynthesis
LIMLP_04680*	LIMLP_RS04670		Sodium:bile acid symporter (putative taurine symporter)	1.035	2.948	Taurine catabolism
LIMLP_04685*	LIMLP_RS04675	*tauD*	Taurine dioxigenase	1.031	3.094	Taurine catabolism
LIMLP_17840*	LIMLP_RS17800	*hemA*	Glutamyl-tRNA reductase	NA	1.006	Heme biosynthesis
LIMLP_17845	LIMLP_RS17805	*hemC*	Uroporphyrinogen synthase	NA	1.027	Heme biosynthesis
LIMLP_03360*	LIMLP_RS03360		UDP-N-acetylglucosamine--peptide N-acetylglucosaminyltransferase, tetratricopeptide repeat protein	NA	1.010	
LIMLP_03380	LIMLP_RS03380	*tmk*	Thymidylate kinase	NA	1.003	Purine and pyrimidine metabolism
LIMLP_04130	LIMLP_RS04120		kua-ubiquitin conjugating enzyme hybrid localization domain protein	NA	1.126	Lipid transport and metabolism
LIMLP_10895*	LIMLP_RS10875		Transkelotase (carbohydrate metabolism)	NA	1.031	Pentose phosphate pathway
LIMLP_10900*	LIMLP_RS10880		Transkelotase (carbohydrate metabolism)	NA	1.137	Pentose phosphate pathway
LIMLP_10910*	LIMLP_RS10890		UDP-glucose 4-epimerase (exposed polysaccharide colonic acid synthesis)	NA	1.201	Carbohydrate biosynthesis
LIMLP_10915*	LIMLP_RS10895		ADP-heptose synthase, Cell wall/membrane/envelope biogenesis (exposed polysaccharide colonic acid synthesis)	NA	1.388	Coenzyme transport and metabolism

LIMLP_10920*	LIMLP_RS10900		mannose-1-phosphate guanylyltransferase (exposed polysaccharide colonic acid synthesis)	NA	1.206	Carbohydrate biosynthesis
LIMLP_10925*	LIMLP_RS10905		NAD-dependent dehydratase (exposed polysaccharide colonic acid synthesis)	NA	1.493	Carbohydrate biosynthesis
LIMLP_10930*	LIMLP_RS10910		phosphoheptose isomerase (carbohydrate metabolism)	NA	1.278	Carbohydrate biosynthesis
LIMLP_14810	LIMLP_RS14775	*yafJ*	Glutamine amidotransferase	NA	1.020	Purine nucleotide biosynthesis
**Regulator**						
LIMLP_03625	LIMLP_RS03620		Anti-sigma factor antagonist	NA	1,144	
LIMLP_05055*	LIMLP_RS05045		Transcriptional regulator MolR	NA	1.276	
LIMLP_05600	LIMLP_RS05595		Transcriptional regulator SutR	NA	1.649	
LIMLP_09440	LIMLP_RS09425		Transcriptional regulator	NA	1.169	
LIMLP_10945*	LIMLP_RS10925		Transcriptional regulator MarR family (exposed polysaccharide colonic acid synthesis)	NA	1.238	
LIMLP_18795	LIMLP_RS18755		fecR/PupR domain containing protein, LipL45-related protein, sigma factor	NA	1.084	
**Oxidative stress**						
LIMLP_02795*	LIMLP_RS02795	*ccp*	Cytochrome C peroxidase	1.663	3.844	
LIMLP_05955*	LIMLP_RS05945	*ahpC*	Peroxiredoxin	NA	2.117	
LIMLP_10145*	LIMLP_RS10130	*katE*	Catalase	NA	1.110	
LIMLP_10155*	LIMLP_RS10140	*perRA*	Transcriptonal regulator Fur family	NA	1.945	
LIMLP_05620*	LIMLP_RS05615	*perRB*	Transcriptonal regulator Fur family	NA	1.233	
**Proteotoxic stress**						
LIMLP_10060*	LIMLP_RS10045	*clpB*	Chaperone Clp ATPase	NA	1.019	
LIMLP_16070	LIMLP_RS16030	*clpC*	Clp ATPase ClpC	NA	1.041	
**Hypothetical**						
LIMLP_00260	LIMLP_RS00260		Hypothetical	NA	1.194	
LIMLP_02105*	LIMLP_RS02105		Hypothetical	NA	1.260	
LIMLP_02260	LIMLP_RS02260		Hypothetical	1.093	2.116	
LIMLP_02425	LIMLP_RS02425		Hypothetical	1.552	4.714	
LIMLP_02935*	LIMLP_RS02935		Hypothetical	2.452	NA	
LIMLP_03640	LIMLP_RS03635		Hypothetical	1.608	2.689	
LIMLP_03685	LIMLP_RS03675		Hypothetical	NA	1.188	
LIMLP_04430	LIMLP_RS04420		Hypothetical	NA	1.970	
LIMLP_04635*	LIMLP_RS04625		Hypothetical (putative lipoprotein)	NA	1.472	

LIMLP_04870*	LIMLP_RS04860		Hypothetical	1.037	2.099	
LIMLP_05015*	LIMLP_RS05005		Hypothetical	NA	1.231	
LIMLP_05115*	LIMLP_RS05110		Hypothetical	1.007	1.770	
LIMLP_05120*	LIMLP_RS23455		Hypothetical	1.112	2.150	
LIMLP_06130	LIMLP_RS06120		Hypothetical	1.012	1.597	
LIMLP_06205*	LIMLP_RS06195		Hypothetical	NA	3.369	
LIMLP_07355	LIMLP_RS07340		Hypothetical	1.024	2.077	
LIMLP_09650*	LIMLP_RS09635		Hypothetical	NA	1.588	
LIMLP_10965*	LIMLP_RS10940		Hypothetical	NA	1.666	
LIMLP_11405*	LIMLP_RS11380		Hypothetical	NA	1.138	
LIMLP_12300	LIMLP_RS12275		Hypothetical	NA	1.150	
LIMLP_13345	LIMLP_RS13315		Hypothetical	NA	1.063	
LIMLP_15890	LIMLP_RS15850		Hypothetical (putative lipoprotein)	NA	1.188	
LIMLP_16025*	LIMLP_RS15985		Hypothetical	NA	1.198	
LIMLP_18725*	LIMLP_RS18685		Hypothetical	NA	1.259	
**Miscellaneous**						
LIMLP_01545*	LIMLP_RS01545		chemotaxis protein	NA	1.331	
LIMLP_02420*	LIMLP_RS02420		Lipid transport and metabolism	2.270	5.369	
LIMLP_02430	LIMLP_RS02340		Peptidyl prolyl isomerase (protein folding)	1.583	4.450	
LIMLP_02435	LIMLP_RS02435		HupE/UreJ protein	NA	3.275	
LIMLP_02440	LIMLP_RS02440		TonB-dependent receptor	NA	1.564	
LIMLP_02840	LIMLP_RS02840		Chemotaxis protein	NA	1.016	
LIMLP_04555*	LIMLP_RS04545		Phosphohydrolase	NA	1.192	
LIMLP_06870*	LIMLP_RS06860		Tetratricopeptide repeat protein	NA	1.238	
LIMLP_07260	LIMLP_RS07245	*lmtA*	Methyltransferase	NA	1.152	
LIMLP_11880	LIMLP_RS11855		Permease (drug exporter, Eam-like)	NA	1.074	
LIMLP_12510*	LIMLP_RS12480		tetratricopeptide repeat protein	NA	1.505	
LIMLP_13380	LIMLP_RS13350		Adenylate cyclase	NA	1.123	Signal transduction
LIMLP_14055	LIMLP_RS14025		Penicilline-binding protein	NA	1.068	
LIMLP_15405	LIMLP_RS15365	*ligA*	Lipoprotein LigA	NA	1.595	
LIMLP_15415	LIMLP_RS15375	*ligB*	Lipoprotein LigB	NA	1.063	
LIMLP_15535*	LIMLP_RS15495	*amtB*	Ammonium channel (transporter)	NA	1.157	
LIMLP_15540*	LIMLP_RS15500	*glnK*	nitrogen regulatory protein P-II	NA	1.206	
LIMLP_16520*	LIMLP_RS16480		DNA repair exonuclease	NA	1.330	

Table S6: Upregulated genes upon exposure to superoxide.

Genes upregulated upon 1 h exposure to 50 μM paraquat.

[a] Gene numeration is according to Satou et al. (2015).

[b] Gene numeration is according to NCBI reference sequence (NZ_CP011931.1, NZ_CP011932.1, NZ_CP011933.1)

[c] Log_2FC (WT exposed to paraquat vs WT) with a $Log_2FC > 1$ and *padj* < 0.05.
* Genes significantly upregulated by H_2O_2 ($Log_2FC > 1$, *padj* < 0.05).

Old locus[a]	New locus[b]	Gene	Function	Log$_2$FC 10 μM PQ[c]	Log$_2$FC 50 μM PQ[c]	
**Metabolism**						
LIMLP_02490**	LIMLP_RS02490		Glycerate lipase	NA	-1.052	Triacylglycerol degradation
LIMLP_05260**	LIMLP_RS05255	*mauG*	Cytochrome C peroxidase, methylamine utilization protein	NA	-2.117	
LIMLP_06070**	LIMLP_RS06060	*atpG*	ATP F0F1 synthase subunit gamma	NA	-1.026	Respiratory chain
LIMLP_06075**	LIMLP_RS06065	*atpD*	ATP F0F1 synthase subunit beta	NA	-1.173	Respiratory chain
LIMLP_06080**	LIMLP_RS06070	atpC	ATP synthase F0F1 subunit epsilon	NA	-1.042	Respiratory chain
LIMLP_06970	LIMLP_RS06960	*pgsC*	capsular biosynthesis protein (AA biosynthesis)	NA	-1.544	
LIMLP_07110*	LIMLP_RS07100		glycosyl transferase	NA	-1.195	Cell wall, membrane biosynthesis
LIMLP_09955	LIMLP_RS09940	metW	homoserine O-acetyl-transferase	NA	-1.053	Methionine biosynthesis
LIMLP_10020**	LIMLP_RS10005		1-aminocyclopropane-1-carboxylate deaminase, D-cysteine desulfhydrase, PLP-dependent	NA	-1.266	Sulfur metabolism
LIMLP_10990**	LIMLP_RS10965		Cytochrome oxidase biogenesis protein	NA	-1.081	
LIMLP_12205**	LIMLP_RS12180	*cmk*	cytidylate kinase	NA	-1.373	Purine and pyrimidine metabolism
LIMLP_12210**	LIMLP_RS12185	*aroA*	3-phosphoshikimate 1-carboxyvinyltransferase	NA	-1.366	Chorismate biosynthesis
LIMLP_18450	LIMLP_RS18410		phosphoglycerate kinase	NA	-1.177	
LIMLP_18460*	LIMLP_RS18420	*cobDQ*	Cobalamin biosynthesis protein CobQ, putative Thr decarboxylase	NA	-1.688	Cobalamin biosynthesis
LIMLP_18465**	LIMLP_RS18425	*cobU*	Adenosylcobinamide kinase	NA	-1.704	Cobalamin biosynthesis
LIMLP_18475**	LIMLP_RS18435	*cbiA*	Cobyrinic acid a,c-diamide synthase	NA	-1.492	Cobalamin biosynthesis
LIMLP_18480**	LIMLP_RS18440	*cobO*	Cob(I)yrinic acid a c-diamide adenosyltransferase	NA	-1.413	Cobalamin biosynthesis
LIMLP_18485**	LIMLP_RS18445	*cobM*	Precorrin-4 C11-methyltransferase	NA	-1.226	Cobalamin biosynthesis
LIMLP_18490**	LIMLP_RS18450	*cobJ*	Precorrin-3B C17-methyltransferase	NA	-1.107	Cobalamin biosynthesis
LIMLP_18495**	LIMLP_RS18455	*cbiJ*	Cobalamin biosynthesis protein	NA	-1.223	Cobalamin biosynthesis
LIMLP_18500**	LIMLP_RS18460	*cobI*	Precorrin-2 C20-methyltransferase	NA	-1.131	Cobalamin biosynthesis
**Translation**						
LIMLP_00830**	LIMLP_RS00830	*mnmE*	tRNA modification GTPase TrmE	NA	-1.083	
LIMLP_01960	LIMLP_RS01960		tRNA Pro	NA	-1.003	
LIMLP_03290**	LIMLP_RS03290		rRNA methyltransferase	NA	-1.102	
LIMLP_03365*	LIMLP_RS03365		tRNA Arg	NA	-1.131	
LIMLP_04350	LIMLP_RS04340		tRNA Thr	NA	-1.938	
LIMLP_11290	LIMLP_RS11265		tRNA Met	NA	-1.227	
**Regulation**						
LIMLP_00575	LIMLP_RS00575		Transcriptional regulator MolR	NA	-1.180	

LIMLP_05330	LIMLP_RS05325		Transcriptional regulator MarR family	NA	-1.020	
LIMLP_12905*	LIMLP_RS12875		Transcriptonal regulator FmdB family	NA	-1.155	
**Miscellaneous**						
LIMLP_00495	LIMLP_RS00495		Hydrolase, metal beta-lactamase domain	NA	-1.041	
LIMLP_00820**	LIMLP_RS00820	*yidC*	preprotein translocase subunit YidC	NA	-1.202	
LIMLP_00825**	LIMLP_RS00825		Jag RNA-binding protein	NA	-1.083	
LIMLP_07420**	LIMLP_RS07405		chemotaxis protein CheY	NA	-1.214	
LIMLP_07425**	LIMLP_RS07410		chemotaxis protein CheD	NA	-1.020	
**Hypothetical**						
LIMLP_02010**	LIMLP_RS02010		Hypothetical (putative lipoprotein)	-2.470	-2.656	
LIMLP_04260	LIMLP_RS04250		Hypothetical	-1.167	-2.402	
LIMLP_04265**	LIMLP_RS22510		Hypothetical	-2.271	-1.982	
LIMLP_04860	LIMLP_RS04850		Hypothetical	NA	-1.077	
LIMLP_04895	LIMLP_RS04885		Hypothetical	NA	-1.391	
LIMLP_05250**	LIMLP_RS05245		Hypothetical	NA	-1.825	
LIMLP_05255**	LIMLP_RS05250		Hypothetical (putative lipoprotein)	NA	-2.144	
LIMLP_05265**	LIMLP_RS05260		Hypothetical	NA	-1.648	
LIMLP_07060	LIMLP_RS07050		Hypothetical	-1.013	-1.283	
LIMLP_08240	LIMLP_RS08225		Hypothetical	-1.732	-3.531	
LIMLP_09385	LIMLP_RS09370		Hypothetical	NA	-1.229	
LIMLP_09780*	LIMLP_RS09765		Hypothetical	-1.123	-2.287	
LIMLP_11240**	LIMLP_RS11215		Hypothetical	NA	-1.424	
LIMLP_11445*	LIMLP_RS11420		Hypothetical	NA	-1.796	
LIMLP_11685**	LIMLP_RS11660		Hypothetical	NA	-1.010	
LIMLP_12910**	LIMLP_RS12880		Hypothetical	NA	-1,323	
LIMLP_14275	LIMLP_RS14240		Hypothetical	NA	-1.209	
LIMLP_15390	LIMLP_RS15350		Hypothetical	NA	-1.594	
LIMLP_15440	LIMLP_RS15400		Hypothetical	-1.100	NA	
LIMLP_15985	LIMLP_RS15945		Hypothetical	NA	-1.086	
LIMLP_16460	LIMLP_RS16420		Hypothetical	NA	-1.881	
LIMLP_16925**	LIMLP_RS16885		Thioesterase	NA	-1.058	
LIMLP_17465**	LIMLP_RS17425		Hypothetical (putative lipoprotein)	NA	-1.195	
LIMLP_18455	LIMLP_RS18415		Hypothetical	NA	-1.723	
LIMLP_18470**	LIMLP_RS18430		Hypothetical	NA	-1.603	
NA	LIMLP_RS20050		Hypothetical	-1.497	-2.240	
NA	LIMLP_RS20165		Hypothetical	-1.038	NA	
NA	LIMLP_RS20565		Hypothetical	NA	-1.498	
NA	LIMLP_RS20630		Hypothetical	-1.272	-1.270	
NA	LIMLP_RS22035		Hypothetical	NA	-1.025	

NA	LIMLP_RS22720		Hypothetical	-1.004	NA	
NA	LIMLP_RS23530		Hypothetical	-1.057	NA	

Table S7: Downregulated genes upon exposure to superoxide.

Genes downregulated upon 1 h exposure to 10 or 50 μM paraquat.

[a] Gene numeration is according to Satou et al. (2015).

[b] Gene numeration is according to NCBI reference sequence (NZ_CP011931.1, NZ_CP011932.1, NZ_CP011933.1)

[c] Log_2FC (WT exposed to paraquat vs WT) with a $Log_2FC < -1$ and *padj* < 0.05.

* Genes significantly upregulated by H_2O_2 ($Log_2FC > 1$, *padj* < 0.05).

** Genes significantly down-regulated by H_2O_2 ($Log_2FC < -1$, *padj* < 0.05).
