# Supplementary material for: Revisiting oxygen toxicity: evolution and adaptation to superoxide in a SOD-deficient bacterial pathogen
Source: mBio. 2025 Jul 23;16(8):e00645-25. doi: 10.1128/mbio.00645-25 (PMC12345160; doi:10.1128/mbio.00645-25)
Supplement: Tables S10 and S11 — Strains, plasmids, and primers used in this study. [file mbio.00645-25-s0009.pdf]

Plasmids	Description [a]	Source
pMaORI	Replicative and conjugative vector for *Leptospira*<br>Resistance to spectinomycin	Pappas et al. (2015)
pSGH1	pMaORI containing the LEPBIa0027 (*sodB*) ORF<br>Promoter: groES<br>Resistance to spectinomycin	This study
pSGH11	pMaORI containing the LEPBIa2495 (*katG*) ORF<br>Promoter: lipL32<br>Resistance to spectinomycin	This study
pSGH10	pMaORI containing the LEPBIa0027 (*sodB*) and LEPBIa2495 (*katG*) ORF<br>Promoter: lipL32 (*katG*) and groES (*sodB*)<br>Resistance to spectinomycin	This study

Strains	Description [b]	Tn insertion site [c]	Source
Leptospira interrogans<br>serovar Manilae, strain L495	Wild-type strain (WT)	NA	
Strain L495 Clone 3	Clone isolated from L495 on EMJH plate	NA	This study
LIMLP_14460 (Man815)	Himar1 Tn insertion in LIMLP_14460<br>LIMLP_14460*::*Km^R<br>Resistance to kanamycin	3425389	Murray et al. (2009)
leuA1 (Man831)	*Himar1* Tn insertion in LIMLP_08570<br>*leuA1::*Km^R<br>Resistance to kanamycin	2075388	Murray et al. (2009)
leuA2 (Man765)	*Himar1* Tn insertion in LIMLP_15720<br>*leuA2::*Km^R<br>Resistance to kanamycin	3757461	Murray et al. (2009)
metY (Man513)	*Himar1* Tn insertion in LIMLP_09200<br>*metY::*Km^R<br>Resistance to kanamycin	2220101	Murray et al. (2009)
Π1 (Δ*thyA*)	*Escherichia coli* replicative strain		
β2163 (Δ*dapA*)	*Escherichia coli* conjugative strain		Demarre *et al.* (2005)

[a] Gene name is according to *Leptospira biflexa* serovar Patoc strain Patoc 1 genome; [b] Gene name is according to *Leptospira interrogans* serovar Manilae strain UP-MMC-NIID-LP genome (Satou *et al.* (2015)); [c] Tn position is according to *Leptospira interrogans* serovar Manilae strain UP-MMC-NIID-LP genome (Satou *et al.* (2015)).

Table S10. Plasmids and strains used in this study

Primer name	Target	Sequence
sodB_qRTF	*sodB*	AACTCCCAGAACTTCCTTATG
sodB_qRTR	*sodB*	ATGCACTTTCAAATTCAGTCC
LEPBIa2495F	*katG*	CTGTTCCTGCCGTAAACCAT
LEPBIa2495R	*katG*	AATCCTTGCTCCATTTGCAC
ori_qRTF	*oriC*	CCTTTTCATCTCCACCCAAA
ori_qRTR	*oriC*	ATTTGTAGCATAACTTTACCCATAA
maoEco1	pMaORI	GGCGCCTCCTTTTGATACTG
maoEco2	pMaORI	CGGGCAGGATAGGTGAAGTA
pMaORI-A	pMaORI	AGATGATCTTCTTGAGATCG
Mao2	pMaORI	ATTCCACATATATTCTGTCC
sodB_F	*sodB*	CCCATATGGAACATAAACTCCCAGAACTTC
sodB_R	*sodB*	CCTCTAGATTAAAGATTTTTATTTGCGAAATCCCAATTCAC
flaB_qRTF	*flaB*	GCCAATGACTCTATCGGGGT
flaB_qRTR	*flaB*	CATTGCTGTTCCCGACTGAA

Table S11: Primers used in this study
